# Characterizing Interlayer Excitons by Spectral Signature in Scattering Visible Near-Field Microscopy

**DOI:** 10.1021/acs.jpclett.5c01052

**Published:** 2025-06-30

**Authors:** Oisín Garrity, Iris Niehues, Annika Bergmann-Iwe, Anna Wróblewska, Luka Pirker, Adeel Bukhari, Gregor Hlawacek, Tobias Korn, Otakar Frank, Patryk Kusch

**Affiliations:** † Department of Physics, 9166 Freie Universität Berlin, Arnimallee 14, D-14195 Berlin, Germany; ‡ Institute of Physics, 9185 University of Münster, 48149, Münster, Germany; § Institute of Physics, 538709 Universität Rostock, 18059 Rostock, Germany; ∥ Faculty of Physics, 49566 Warsaw University of Technology, Koszykowa 75, 00-662, Warsaw, Poland; ⊥ 48311 J. Heyrovský Institute of Physical Chemistry of the CAS, Dolejškova 2155/3, 182 00 Prague 8, Czech Republic; # Faculty of Mathematics and Physics, 37740 Charles University, Ke Karlovu 3, 12116, Prague, Czech Republic; ∇ Helmholtz-Zentrum Dresden−Rossendorf, 28414 Institut für Ionenstrahlphysik und Materialforschung, D-01328 Dresden, Germany

## Abstract

Interlayer excitons (IXs) in van der Waals heterostructures exhibit unique optical properties due to their spatially separated charge carriers. However, the weak oscillator strength and radiative broadening of IXs make them difficult to detect with conventional absorption spectroscopy. Here, we use scattering-type scanning near-field optical microscopy (s-SNOM) to directly probe the dielectric response at the nanoscale. We first validate this approach by measuring the B-exciton in a four-layer MoS_2_ sample, where ion irradiation introduced defect-induced broadening. Extending this method to a MoSe_2_/WSe_2_ heterostructure, we observe a Lorentzian resonance at 1.35 eV, characteristic of interlayer excitons, with broadening dominated by nonradiative decay. These results demonstrate the capability of s-SNOM to image and characterize weak excitonic resonances at the nanoscale, overcoming the limitations of conventional techniques and providing new insights into localized exciton dynamics in 2D heterostructures.

## Graphical abstract

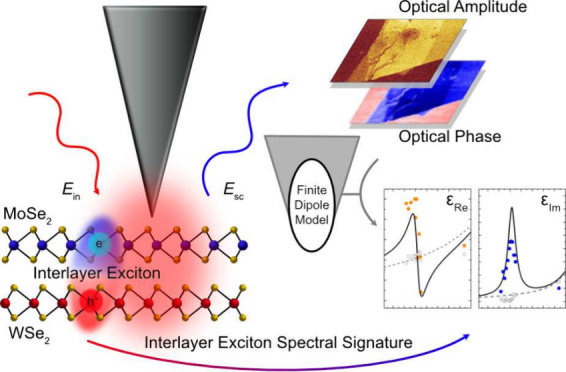

Two-dimensional (2D) transition-metal dichalcogenides (TMDCs) have attracted considerable interest for excitonic research, because their large exciton binding energies remain stable at room temperature. Monolayer TMDCs exhibit direct bandgaps and exciton-dominated optical responses, which can be tuned optically, electrically, or mechanically.^1,2^ Stacking two different TMDC monolayers often leads to staggered band alignment and the formation of interlayer excitons (IXs) with lower oscillator strength and longer lifetimes than intralayer excitons.^3,4^

Interlayer excitons (IXs) have emerged as exciting platforms for fundamental studies and novel optoelectronic devices. For example, IXs in MoSe_2_/hBN/MoSe_2_ heterostructures can be electrically tuned, exhibit diffusion lengths >10 μm, and feature line widths <4 meV.^5^ Their properties open avenues to many-body phenomena (e.g., condensates and superfluidity) and enable photon antibunching for single-photon emitters—promising for quantum electronics. An hBN spacer additionally facilitates electrical control and diffusion of IXs in heterostructures.^6–8^

Advanced spectroscopic techniques (e.g., photoluminescence (PL), absorption, pump–probe) probe IX transition energies and dynamics.^9^ Although tip-enhanced PL can surpass the diffraction limit,^10^ it offers limited quantitative information. In contrast, scattering-type scanning near-field optical microscopy (s-SNOM) accesses the dielectric function at nanometer resolution.^11,12^ Despite success on 2D materials, s-SNOM has yet to be applied to interlayer excitons at their transition energies.

In this work, we apply s-SNOM to directly probe the spectral signature of interlayer excitons (IXs) in MoSe_2_/WSe_2_ heterostructures. These excitons possess out-of-plane dipole moments and long lifetimes, making them uniquely sensitive to the local dielectric environment. However, their spatial separation and low oscillator strength pose a challenge for detection by using conventional optical techniques. We demonstrate that s-SNOM can spatially resolve the near-field response of IXs with nanometer resolution, offering a new pathway to investigate their optical signatures and dynamic properties.

To validate our methodology, we first apply it to a pristine monolayer of MoS_2_ on hBN, where the extracted exciton parameters from s-SNOM closely match those from PL and literature values (see Supporting Information - Figure S2). We then turn to a four-layer MoS_2_ slab, acquiring a series of s-SNOM images across the B-exciton (XB^M^) resonance and plotting the response as a function of the excitation energy. Photoluminescence (PL) spectroscopy provides key parameters for exciton decay, which are incorporated into a dielectric function model based on a Lorentz oscillator.^13,14^ This function is then used within a multilayer finite-dipole framework^15^ to simulate the near-field response, yielding a good fit to the measured spectra. This procedure successfully retrieves the known optical response of XB^M^ in MoS_2_ and establishes a robust basis for extracting dielectric functions from near-field data while allowing us to explore the impact of disorder and broadening in the multilayer case.

Applying the same approach to the MoSe_2_/WSe_2_ heterostructure, we identify the IX transition via PL and fit the near-field spectra using the finite-dipole model.^15^ The resulting dielectric function reveals a resonance with small radiative and large nonradiative line widths—signatures typical of IXs at room temperature. These results demonstrate the ability of s-SNOM to detect, image, and characterize interlayer excitons on the nanoscale, offering valuable insights into their optical and decay properties. To realize this, we fabricated and characterized TMDC heterostructures by using a combination of optical spectroscopy and near-field microscopy, described in the following.

SiO_2_/Si substrates with a thickness of 300 nm were cleaned by sonication in acetone and IPA followed by an O_2_ plasma cleaning. An MoS_2_ crystal (flux grown, 2D semiconductors) was freshly cleaved via mechanical exfoliation using tape^16^ and pressed on clean substrates. The substrates were then annealed at 65 °C for 6 h. After being cooled to room temperature, the samples were rinsed in acetone to remove any residues.

Single monolayers of WSe_2_ and MoSe_2_ were isolated by mechanical exfoliation and transferred to a polydimethylsiloxane (PDMS) stamp.^16^ The WSe_2_ monolayer was stamped onto a Si/SiO_2_ substrate with the MoSe_2_ monolayer then stamped on top, and observed in situ via optical microscopy. To achieve reciprocal space alignment and optimize interlayer exciton photoluminescence yield, the straight edges of each monolayer were used to estimate the twist angle with the aim of achieving either zero or 60 degree interlayer twist.^17^ Following the transfer, the heterostructure was placed in a test tube which was then submerged in an oil bath at 300 °C for 4 h at a pressure of 2 × 10^–7^ mbar. This method facilitates the removal of polymer residues and reduces interfacial bubbles through capillary action and thermal relaxation.^18^ No AFM was performed on this specific region; however, visual inspection of the near-field phase and amplitude maps (Supporting Information, Figure S5­(a,d)) revealed a reduction in bubble density following annealing of the heterostructure. The RMS surface roughness in the active region was measured to be approximately 974 pm (surface slope = 26.2 × 10^–3^, Figure S5­(b)).^19^ Room-temperature PL characterization was performed using a Horiba Jobin-Yvon XploRA micro-Raman spectrometer with a 0.90NA (NA: numerical aperture) 100× objective. The excitation laser was 532 nm with 1 mW power, acquisition time of 1 s, and a 600 grooves per mm grating.

The 4L-MoS_2_ was analyzed using a dual s-SNOM system^11,20^ (NeaSNOM from attocube systems AG, Germany) integrated with nano-FTIR tips (attocube systems AG) that had an apex radius ≈ 50 nm and a resonance frequency in the range 240–380 kHz. For 1L-MoS_2_ and the MoSe_2_/WSe_2_ heterostructure, a PtIr-coated tapping-mode tip (NanoWorld Arrow-NCPt, nominal radius ≈ 20 nm) was used. The resolution of these near-field probes has been revealed in previous work to be 20 nm.^21^

The excitation source was a continuous wave (cw) laser (Hübner C-Wave) tunable across multiple spectral bands: 450–525 nm, 540–650 nm, 900–1050 nm, and 1080–1300 nm, corresponding to photon energies of approximately 2.76–2.38 eV, 2.30–1.91 eV, 1.38–1.18 eV, and 1.15–0.95 eV, respectively. The laser beam was directed through a beam expander and then, via side-illumination, focused at an angle of 45° to the sample by a parabolic mirror, 0.70NA, onto an AFM tip. This allows the tip to function as a near-field probe in the visible range with nanometer precision. For s-SNOM imaging, a laser power of around 1 mW was maintained at the tip with an integration time of 10 ms and a polarization along the tip shaft (p-polarized). The same parabolic mirror also collected the backscattered light and was guided to a single-line silicon CCD detector. The AFM tip operated in tapping mode with an amplitude of 61 nm at a frequency of 267 kHz allowing demodulation of the signal at higher harmonics via lock-in detection and pseudoheterodyne interferometry. The former isolates the near-field contribution through tip-modulated harmonics, while the latter enables accurate phase and amplitude extraction by modulating the reference mirror in an interferometric detection scheme.^22^

To extract the spectral response of excitons, we analyze raw s-SNOM amplitude and phase images acquired as a function of excitation wavelength, spanning the XB^M^ (≈2 eV) and IX (1.35 eV) resonances. To suppress far-field artifacts and enhance the near-field signal, we compute contrast ratios at different harmonics — specifically, *s*_3_/*s*_2_ for amplitude and ϕ_4_ – ϕ_3_ for phase — normalized to the stable spectral response of the SiO_2_/Si substrates. Representative images are provided in the Supporting Information.^23,24^ This procedure enables direct access to the complex dielectric function and reveals the spectral signatures of the excitonic resonances.

The absorption/reflection measurements were carried out at room temperature via a microabsorbance setup built on site.^25^ For excitation, we used a supercontinuum broadband laser (NKT-FIU 15). The light was guided into an inverted microscope (Olympus, IX71) and through a 0.90NA 100× objective, where it is either reflected (*R*) or transmitted (*T*). The laser spot sample position was visible through a microscope camera and mounted on an *xy* translation stage. The transmitted light (*T*) was collected with a 0.80NA 100× objective and guided to an Avantes spectrometer through an optical fiber. The reflected light (*R*) was collected via an additional beam splitter and collimated with a lens onto an optical fiber and onto the same Avantes spectrometer. Absorbance (*A*) was calculated using *A* = 100% – *R* – *T*.^26^

HIM is a versatile method for modifying and imaging a wide range of materials, including insulating and biological materials. A Carl Zeiss OrionNanoFAB setup was used in this work for all the MoS_2_ samples. It allowed the local irradiation of individual flakes using a focused He+ ion beam with a diameter of only 0.5 nm. The energy of the ions used was 7.5 keV. Transfer characteristics were recorded in situ at different steps of fluences with the help of an Agilent Parameter Analyzer (Agilent 4156C Precision Semiconductor Parameter Analyzer). With the samples and optical setup prepared, we now turn to near-field analysis of the excitonic resonances.

While s-SNOM has previously been used to investigate excitons in TMDCs,^27^ its sensitivity to weak optical resonances such as IXs remains unexplored. Our goal is to demonstrate that s-SNOM can detect and resolve the spectral signature of IXs from the dielectric function at the nanoscale. To establish the reliability of our method, we first apply it to a well-characterized four-layer MoS_2_ slab, Figure 1(a), where the XB^M^ exciton provides a strong and well-defined spectral feature in the visible range. In parallel, we validate the multilayer dielectric model itself using pristine monolayer MoS_2_ on hBN, where the XB^M^ line shape is sharp and well-documented (see Supporting Information Figure S2, Table S1). These benchmarks allow us to validate both the optical model and our s-SNOM acquisition and processing strategy and to explore the influence of defects on the exciton response, before turning to the more challenging case of IXs in MoSe_2_/WSe_2_ heterostructures.

**Figure 1:**
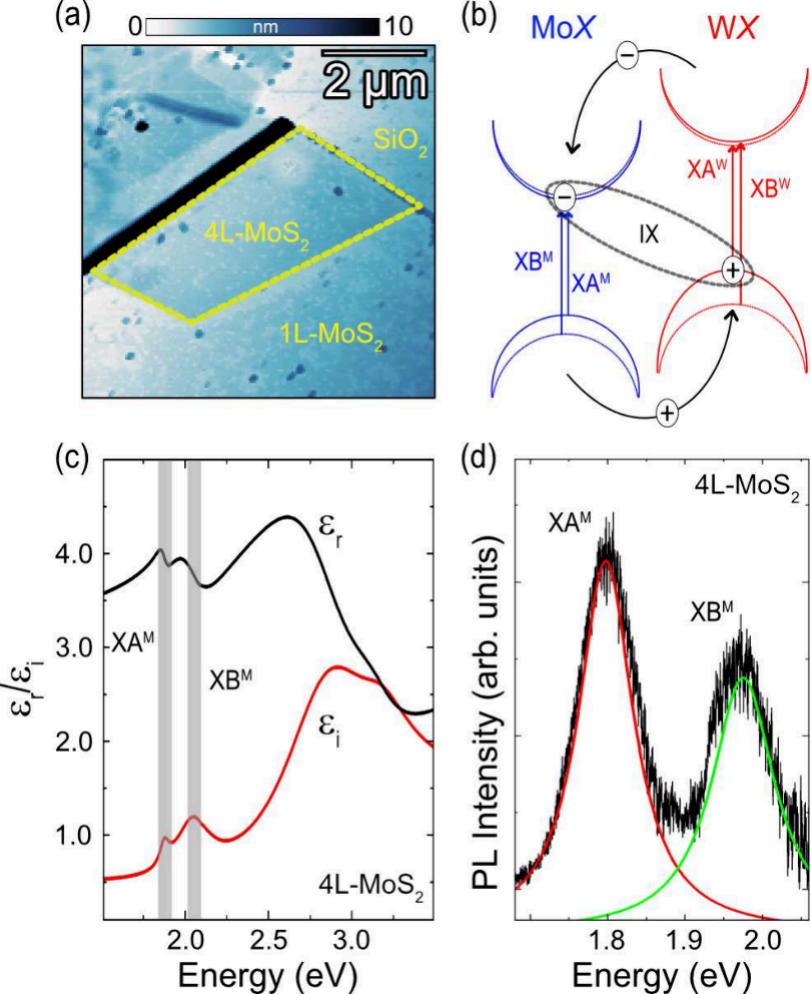
(a) s-SNOM AFM topography image of the 4L-MoS_2_ (yellow border), surrounded by 1L-MoS_2_ and SiO_2_ substrate. (b) The staggered band gap alignment showing momentum direct transitions from the intralayer excitons XA^*M*^ (blue - MoSe_2_) and XA^*W*^ (red - WSe_2_), and the momentum in-direct transition from the interlayer exciton IX. (c) The dielectric function of 4L-MoS_2_, with the real part, ϵ_*r*_, and the imaginary part ϵ_*i*_, with the gray bar indicating the XA^*M*^ and then XB^*M*^ exciton resonances. (d) PL spectra of the 4L-MoS_2_ showing emission from the XA^*M*^ at 1.80 eV and from the XB^*M*^ at 1.97 eV.

In monolayer form, MoS_2_ is a direct band gap semiconductor that hosts two prominent intralayer excitons, XA^M^ and XB^M^, as illustrated in the band structure diagram of Figure 1(b) (blue bands). While the global band structure changes to an indirect gap with bilayers and higher layer numbers, these excitonic resonances still significantly influence the dielectric function, shown in Figure 1(c), and give rise to a measurable photoluminescence (PL) response in Figure 1(d).

To investigate the impact of defect-induced broadening on exciton dynamics, the 4L-MoS_2_ slab was subjected to ion irradiation with a fluence of 5.95 × 10^14^ ions/cm^2^, introducing defect centers that enhance nonradiative recombination pathways and broaden excitonic resonances. This controlled disorder allows us to study how inhomogeneous broadening affects exciton behavior in a realistic defect-rich environment.

To access the spectral signature of the XB^M^ exciton in 4L-MoS_2_, we perform two steps: (i) normalize s-SNOM measurements to the SiO_2_ substrate across the excitation spectrum and (ii) model the near-field interaction between the tip and the sample. Existing near-field models are often limited to the infrared range, assume bulk coupling, or simplify the tip as a point dipole,^20,28,29^ making them unsuitable for layered materials with excitonic transitions in the visible. We therefore adopt the finite-dipole model by Hauer et al.,^15^ which accounts for layered geometries and excitonic resonances, and modify it to account for sharp excitonic transitions in the visible.

Since XA^M^ lies outside our spectral range, we focus on the XB^M^ transition. In s-SNOM, incident light is focused onto a metallic AFM tip, generating a confined near-field at the tip–sample interface. The resulting signal depends on the tip geometry, tip–sample distance, and sample dielectric function.^20^ With an ∼ 50 nm tip apex, we surpass the diffraction limit and access excitonic behavior at the nanoscale. Background subtraction, described in the setup description, separates the detected signal into amplitude (*s*) and phase (ϕ) at higher harmonics of the tip tapping frequency.^22^

Specifically, for the XB^M^ exciton in 4L-MoS_2_ (Figure 1(a)), we analyzed s-SNOM contrast values from amplitude and phase images (Figure 2(a/d – 1.99 eV, b/e – 2.13 eV)) measured over the energy range 1.95–2.35 eV. Amplitude (phase) images were divided (subtracted) by their previous harmonic (*n* – 1) to remove far-field reflection artifacts^23^ and subsequently normalized to the SiO_2_ substrate contrast, Figure 1(a). The average amplitude (phase) contrast of 4L-MoS_2_ at each excitation energy is indicated by orange (blue) circles in Figure 2(c/f). The observed contrast variation exhibits a Lorentzian resonance behavior, described by

1 $$\epsilon \left(\omega \right)=\epsilon_{\infty }+\underset{j\in \left\{A,B\right\}}{\sum }\frac{A_{j}}{\omega^{2}-\omega_{j}^{2}+i\Gamma_{j}}$$

where *A*_*j*_ = *f*_*j*_γ_*j*_ is a fitted amplitude parameter, representing the product of the oscillator strength *f*_*j*_ and the radiative line width γ_*j*_. The total line width Γ_*j*_ = γ_*r*_^*j*^ + γ_*nr*_^*j*^ includes contributions from both radiative γ_*r*_ and nonradiative γ_*nr*_ decay. For XB^M^, we use *A*_*B*_ = 10^7^ meV, a value that ensures the simulated near-field contrast remains consistent with the measured amplitude response, even under substantial nonradiative broadening.^30,31^ ω is the excitation frequency, ω_*j*_ denotes the resonance frequency for each excitonic mode (XA^M^, XB^M^), and ϵ_∞_ is the background dielectric constant, previously reported as ≈ 4.5 for MoS_2_.^32^

**Figure 2:**
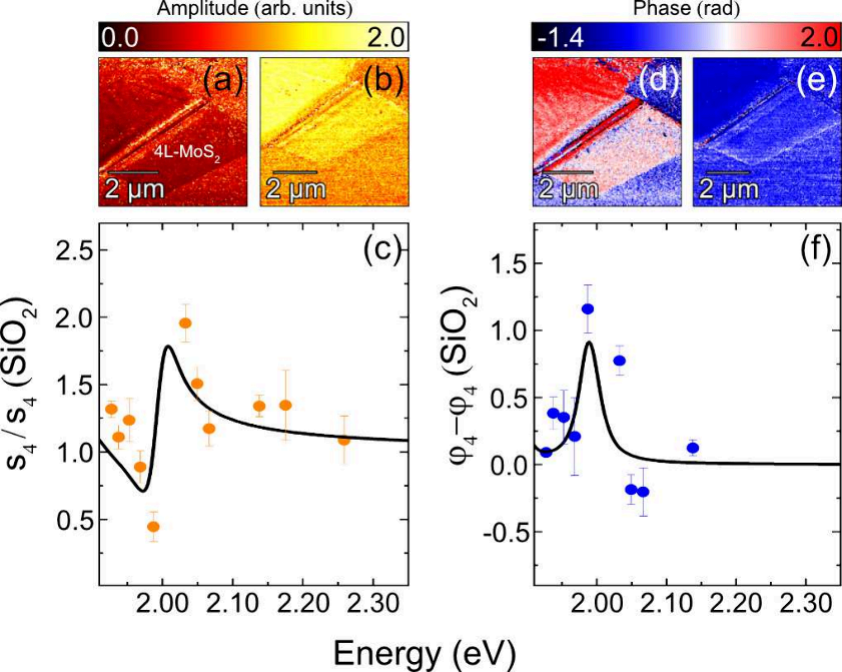
(a,b) s-SNOM amplitude images taken at 1.99 and 2.14 eV, respectively. (c) Fourth harmonic 4L-MoS_2_ amplitude contrast values (s_4_) normalized by the SiO_2_ contrast values (s_4_ (SiO_2_)) (orange circles). (d,e) s-SNOM phase images taken at 1.99 and 2.14 eV, respectively. (f) Fourth harmonic 4L-MoS_2_ phase contrast values (ϕ_4_) normalized by the SiO_2_ contrast values (ϕ_4_ (SiO_2_)) (blue circles). Color-bars show amplitude and phase contrast relative to bare SiO_2_. The black line indicates the fit using eq 1. Error bars represent the standard deviation of the average contrast values.

To accurately extract the resonance parameters, we must account not only for the excitonic Lorentzian response but also for the near-field interactions. We model these interactions using the finite-dipole model, which approximates the AFM tip as a perfectly conducting ellipse and incorporates near-field effects via the method of images (see Supporting Information).^20,33^ The sample structure consists of three optically relevant layers: an air layer, a 4L-MoS_2_ slab (≈3 nm thick), and an SiO_2_ layer, which is treated as semi-infinite for simplicity. To account for the influence of multiple interfaces, we extend the finite-dipole model to layered systems, incorporating the dielectric response functions of each layer and their respective thicknesses.^15^ To validate the accuracy of this approach, we first applied the multilayer FDM model to a pristine 1L-MoS_2_ flake on 10 nm of hBN. The resulting fit reproduced the near-field contrast across the B-exciton resonance with excellent agreement to the PL spectrum and literature values (see Figure S2 and Table S1 - Supporting Information), establishing a reliable reference point for analysis of more complex systems.

Utilizing this model, along with the Lorentzian oscillator model for the two excitonic resonances (XA^M^ and XB^M^) (eq 1), the extracted s-SNOM amplitude (Figure 2(c)) and phase (Figure 2(f)) contrast data were fitted using parameters suggested by the PL response (Figure 1(d)), seen in Table 1.^15,34^ Both the optical amplitude and phase data show good agreement with the fit (black line), clearly highlighting the resonance of the XB^M^ exciton. The influence of the XA^M^ exciton, which could not be directly measured as it lies outside the range of our laser systems, is evident on the left side of Figure 2(c), where an additional Lorentzian oscillator was incorporated to account for its contribution, as described by eq 1.

**Table 1:** Fitting Parameters Extracted Using Eq. 1 for the 4L-MoS_2_ XB^*M*^ Exciton and Eq. 2 for the MoSe_2_/WSe_2_ Heterostructure Interlayer Exciton (IX)^a^

	ω_0_ (meV)	γ_*r*_ (meV)	γ_*nr*_ (meV)	Γ (meV)
4L-MoS_2_ XA^*B*^	2010	20	30	50
MoSe_2_/WSe_2_ HT IX	1355	5	15	20

a Here, *ω*_0_ denotes the resonance energy, while *γ*_*r*_ and *γ*_*nr*_ represent the radiative and non-radiative line widths, respectively. *Γ* is the total line width, given by *Γ* = *γ*_*r*_ + *γ*_*nr*_.

The total line widths, Γ_*B*_ = γ_*r*_^*B*^ + γ_*nr*_^*B*^, of the B exciton in 4L-MoS_2_ were fitted to a value of Γ_*B*_ = 50 meV, exhibiting significant excitonic damping consistent with the introduction of additional recombination channels due to ion irradiation. Photoluminescence (PL) spectroscopy of the same flake shows a clear broadening of the B exciton peak from 30 meV in the pristine region to 132 meV postirradiation, corroborating the disorder-induced damping inferred from the near-field response (See Supporting Information, Figure S3 and Table S2). The total line width from the s-SNOM fit is also consistent with the typical values reported for monolayer MoS_2_ B excitons at room temperature (30–50 meV).^35^ This increase is primarily due to an enhanced radiative line width, as revealed by our fitting model. Specifically, using a Lorentz oscillator model where the numerator includes the product of the oscillator strength and the radiative line width, we fix the radiative and nonradiative components to γ_*r*_^*B*^ = 20 meV and γ_*nr*_^*B*^ = 30 meV, respectively. To match the observed near-field contrast, the fitted oscillator strength parameter was set to *A*_B_ = 2.7 × 10^6^ meV^2^, resulting in a total line width of 50 meV. This high amplitude scaling is consistent with strong near-field coupling in heavily irradiated regions, even when the far-field resonance remains narrow.^27,36^

Having validated the method by successfully recovering the spectral response of the XB^M^ exciton in 4L-MoS_2_, we now apply it to study interlayer excitons (IXs) in a MoSe_2_/WSe_2_ heterostructure (HT), as shown in Figure 3(a). Since WSe_2_ exhibits a higher quantum yield than MoSe_2_, the MoSe_2_ layer (purple, Figure 3(a)) was transferred on top of the WSe_2_ layer (green, Figure 3(a)).^37,38^ The topography in Figure 3(a) reveals interfacial bubbles and wrinkles within the HT region, which are commonly introduced during dry transfer and may contribute to additional nonradiative recombination channels. This is consistent with the significant line width broadening observed in the photoluminescence (PL) spectra in Figure 3(b), where the monolayer emission is quenched and replaced by an IX peak at ∼ 1.35 eV.

**Figure 3:**
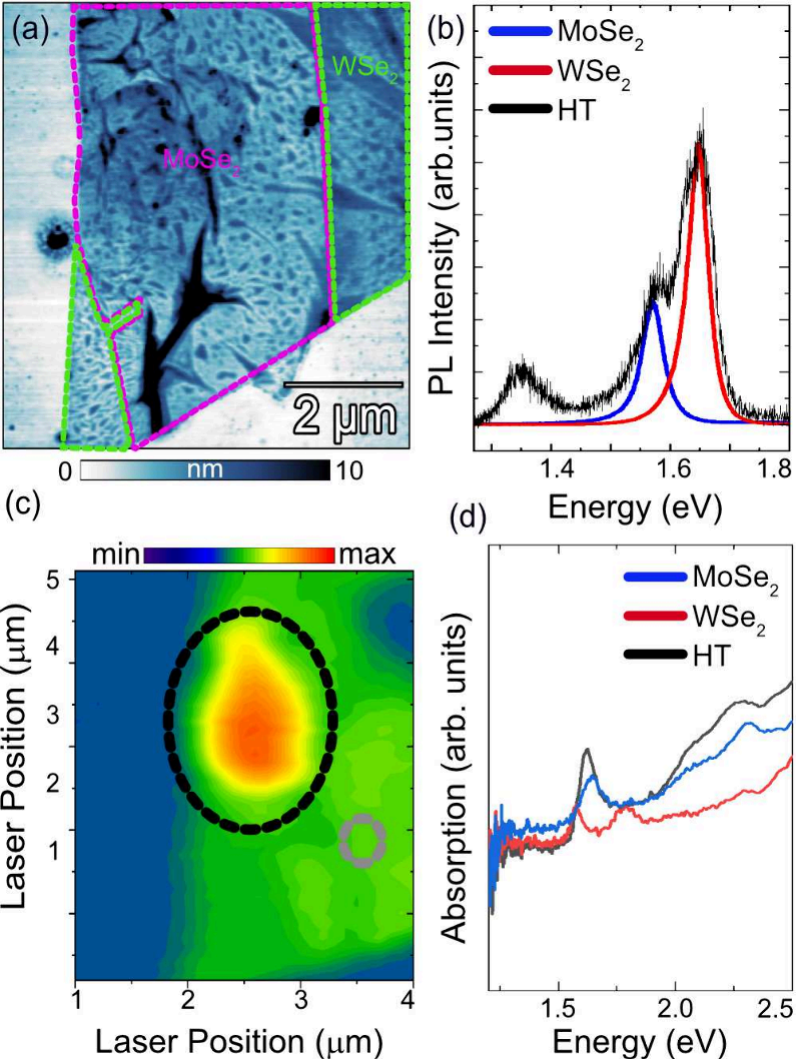
(a) s-SNOM AFM image of the MoSe_2_/WSe_2_ heterostructure, purple for MoSe_2_, green for WSe_2_. (b) PL spectrum with XA^*M*^ (blue) at 1.57 eV, XA^*W*^ (red) at 1.64 eV, and IX (black) at 1.35 eV. (c) PL intensity map at 1.35 eV; black circle indicates measurement area for HT while gray circle indicates control measurement area (WSe_2_). (d) Absorption of 1L of MoSe_2_ (blue), 1L of WSe_2_ (red), and HT (black).

The combination of these two monolayer materials results in a staggered band alignment, as depicted in Figure 1(b), where the conduction band is localized in the MoSe_2_ layer and the valence band resides in the WSe_2_ layer.^39,40^ This electronic structure supports two momentum-direct optical transitions corresponding to the intralayer excitons of MoSe_2_ at 1.57 eV (XA^M^, blue) and WSe_2_ at 1.64 eV (XA^W^, red).^39^ Additionally, an indirect transition arises due to charge transfer, where electrons (holes) migrate from WSe_2_ (MoSe_2_) into MoSe_2_ (WSe_2_), forming an IX. The electron, confined to the MoSe_2_ layer, remains Coulombically bound to a hole in the WSe_2_ layer, establishing a spatially separated IX state.^41^ These electronic and optical properties are reflected in the spectroscopic measurements.

The expected intralayer excitonic transitions appear in the PL spectrum, Figure 3(b), for MoSe_2_ (XA^M^, blue) and WSe_2_ (XA^W^, red) at the previously mentioned peak energies, along with an additional peak at 1.35 eV, attributed to the interlayer exciton (IX).^17,39,41^ A PL intensity map (Figure 3(c)) reveals a spatial region within the heterostructure where IXs are active; however, the resolution is limited by the diffraction limit. Another technique to investigate these transitions is absorption spectroscopy, Figure 3(d), which clearly shows absorption features for the intralayer excitons XA^M^ (blue) and XA^W^ (red), but notably, no absorption from IX is observed due to its indirect transition. Using these two spectroscopic methods, we determine the transition energies of XA^M^ and XA^W^; however, the IX is visible only in PL and is entirely absent in absorption. Additionally, both techniques are limited by diffraction, restricting spatial resolution and rendering them impractical for probing the small active area of interlayer excitons (IXs) in this system. We note that the absorption spectrum was measured on a MoSe_2_/WSe_2_ heterostructure on sapphire, whereas the PL was acquired on a similar heterostructure on a SiO_2_ substrate.

The spectral signatures of the in-plane excitons, XA^M^ and XA^W^, in this heterostructure have been previously measured using s-SNOM. However, the influence of the interlayer exciton (IX) was negligible in earlier studies, as its resonance energy lay outside the spectral range investigated.^27^ To capture the IX’s spectral response from the dielectric function, we extracted s-SNOM contrast values from amplitude and phase images recorded across excitation wavelengths from 1.2 to 1.5 eV, as suggested by the IX PL response in Figure 3(a). Representative images are shown at 1.32 and 1.37 eV in Figure 4(a,d) and Figure 4(b,e), respectively. The contrast evolution across this spectral range was interpreted using the finite dipole model adapted for layered systems with sharp excitonic transitions. Within the ∼ 25 nm resolution of s-SNOM, the IX-active area, previously identified in the PL intensity map (Figure 3(c)), is clearly distinguishable from the surrounding heterostructure, now resolved with a much higher spatial precision. In the phase images (Figure 4(d,e); see Supporting Information), the IX-active region exhibits enhanced contrast before fading as the IX transition energy is crossed. In contrast, the amplitude images (Figure 4(a,b); see Supporting Information) reveal a corresponding decrease in signal amplitude across the resonance.

**Figure 4:**
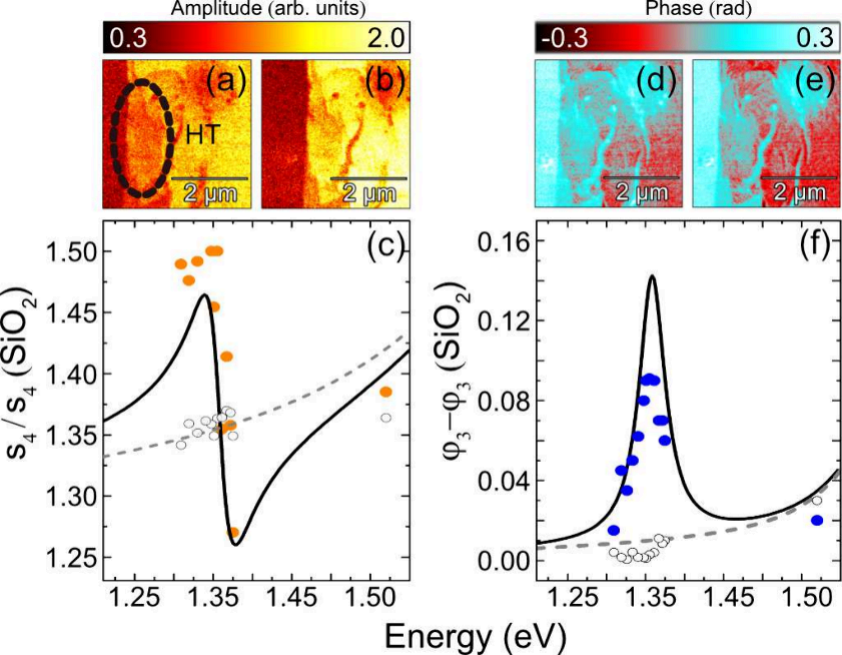
Optical amplitude (a,b) and phase (d,e) images taken at 1.32 and 1.37 eV. Color-bars show amplitude and phase contrast relative to bare SiO_2_. (c) Average amplitude contrast values (orange circles) and (f) phase values (blue circles). Background permittivity of the HT (gray dashed line) was taken from ref 42 and adapted for use here for comparison. Using eq 2 and the finite dipole model for layered systems, a fit (black line) is calculated from the dielectric function of the layered sample (Supporting Information), see Table 1. Phase is referenced to SiO_2_ using the standard pseudoheterodyne convention outlined in ref 22, where only relative phase contrast is accessible. The error bars, calculated from the standard deviation of the contrast in the 4L-MoS_2_ region, are present but too small to be visible.

The average s-SNOM contrast values from the IX-active region (black circle, Figure 3(c) /Figure 4(a)) and the non-IX region (gray circle, Figure 3(c)) were extracted and plotted as a function of excitation energy for both amplitude (Figure 4(c), orange circles) and phase (Figure 4(f), blue circles). The third harmonic demodulation was chosen for the phase signal as it provided higher signal-to-noise and more consistent contrast across the HT region compared to the fourth harmonic. The contrast from the IX-active region exhibits a Lorentzian resonance centered at 1.35 eV, whereas the non-IX region (white circles) shows no such resonance and instead follows the background permittivity of the heterostructure (gray line).^42^

To model the IX resonance, we combine a Lorentz oscillator representation of the dielectric function with the finite-dipole model adapted from Hauer et al.,^15^ modified to describe layered materials with sharp excitonic transitions in the visible spectral range:^13–15^

2 $$\epsilon \left(\omega \right)=\epsilon_{\infty }-\frac{A_{\mathrm{IX}}}{\omega -\omega_{0}+i\Gamma_{\mathrm{IX}}}$$

where *A*_IX_ is a fitted amplitude parameter related to the oscillator strength of the interlayer exciton, and Γ_IX_ is its total line width. All parameters have meaning analogous to those in eq 1, with specific fit values summarized in Table 1. Using eq 2 with the finite dipole model for multilayered systems adapted from Hauer et al., a fit with a single Lorentzian oscillator accurately reproduces the observed near-field contrast, allowing extraction of the exciton resonance energy and associated line width parameters.

Notably, unlike the intralayer exciton in 4L MoS_2_ (positive near-field contrast), the IX exhibits a negative resonance in s-SNOM. This difference arises from the strong out-of-plane dipole moment of IXs, enhancing the dielectric screening and reducing the local near-field response. Additionally, the IX transition is dominated by resonant absorption rather than scattering, leading to suppressed near-field amplitude.

The transition energy of the interlayer exciton (IX), extracted from the fit, ω_0_ = 1.35 meV agrees with the PL peak in Figure 3(a). Although the heterostructure has a near-zero twist angle, the IX is expected to be momentum-indirect (e.g., K-Q), consistent with its weak optical activity. The extracted nonradiative line width, γ_nr_ = 15 meV, is in line with prior reports for such transitions,^4^ while the radiative line width, γ_r_ = 5 meV, reflects the suppressed oscillator strength typical of spatially indirect interlayer excitons.^4^

The large broadening of the IX resonance arises from a combination of intrinsic and extrinsic factors. Intrinsically, the interlayer nature of the exciton leads to a reduced oscillator strength due to spatial separation, while phonon interactions at room temperature further contribute to nonradiative broadening.^43^ However, the HT morphology shows distinct wrinkles and interfacial bubbles, which can contribute to local strain and broadening effects in the near-field response, as shown in Figure 3(a,b). Unlike the majority of the heterostructure, this localized region exhibits strong IX photoluminescence, suggesting that it formed under distinct conditions. Given that the heterostructure was assembled via mechanical exfoliation and annealed at 300 °C under high pressure, it is likely that the layers within this region serendipitously aligned to a favorable interlayer twist angle. Defects, in addition to wrinkles and bubbles seen in Figure 3(a), introduced during fabrication and annealing, may have contributed to stabilizing this alignment while also generating localized strain fields and potential fluctuations. These factors could further inhomogeneously broaden the IX resonance by modifying local band alignment and increasing nonradiative decay pathways. It is also likely that the heterostructure is at an interlayer twist angle of a few degrees. Locally, atomic reconstruction may occur, where the layers subtly deform to form large regions of well-defined interlayer registry. In other areas, a moiré-type lattice persists. Prior studies using Raman and PL spectroscopy^44^ suggest that regions of atomic reconstruction correlate with enhanced IX PL yield.

Similar to the irradiated 4L-MoS_2_ sample, where defect-assisted recombination was a dominant broadening mechanism, the IX-active region may experience enhanced nonradiative decay due to defect-related trap states, further increasing its line width. This highlights the importance of local disorder in shaping the optical response of interlayer excitons, reinforcing the utility of s-SNOM in accessing excitonic resonances that remain undetectable by conventional absorption spectroscopy.

While fitting a Lorentzian model to near-field data can exhibit parameter degeneracy, where multiple combinations of oscillator strength and line width yield comparable results, this ambiguity is significantly mitigated by cross-validation with PL spectroscopy. The line widths and peak energies extracted from the near-field model are consistent with the PL spectra, and the large amplitude parameters required for matching near-field contrast are supported by prior work on resonant nanophotonics in 2D materials.^30,31^ This consistency across the techniques strengthens the reliability of the extracted dielectric response.

We demonstrate that s-SNOM can resolve intra- and interlayer excitons at the nanoscale, providing access to their spectral signatures and transition energies. This is particularly valuable for detecting interlayer excitons, whose weak oscillator strength and spatial separation limit far-field techniques.

As a validation, we recover the B-exciton response in 4L-MoS_2_, including broadening from ion irradiation. Applying the same method to a MoSe_2_/WSe_2_ heterostructure, we resolve the interlayer exciton resonance and fit the contrast using a finite-dipole model modified for layered systems in the visible range.

While the model captures the spectral lineshapes and yields plausible line widths, its current form limits precise lifetime extraction. Future refinements—such as cryogenic measurements or improved dielectric models—could enhance quantitative access to exciton dynamics.

These results establish s-SNOM as a powerful probe of excitonic behavior in complex, twisted, and disordered heterostructures with applications in quantum optoelectronics and 2D materials science.

## Supplementary Material





## Data Availability

The data and custom code that support the findings of this study are available from the corresponding author upon reasonable request.

## Notes

PL and s-SNOM data for 1L- and 4L-MoS_2_; details of finite dipole model implementation; raw and processed contrast maps; AFM roughness and phase correction analysis. Supporting Figures S1–S6 and Tables S1–S2 (PDF)
Transparent Peer Review report available (PDF)

## References

[1] Splendiani A., Sun L., Zhang Y., Li T., Kim J., Chim C.-Y., Galli G., Wang F. (2010). Emerging photoluminescence in monolayer
mos_2_. Nano Lett..

[2] Xiao J., Zhao M., Wang Y., Zhang X. (2017). Excitons in atomically
thin 2D semiconductors and their applications. Nanophotonics.

[3] Mak K. F., Lee C., Hone J., Shan J., Heinz T. F. (2010). Atomically thin
mos_2_: A new direct-gap semiconductor. Phys. Rev. Lett..

[4] Jiang Y., Chen S., Zheng W., Zheng B., Pan A. (2021). Interlayer
exciton formation, relaxation, and transport in tmd van der waals
heterostructures. Light Sci. Appl..

[5] Zhang L., Gu L., Ni R., Xie M., Park S., Jang H., Ma R., Taniguchi T., Watanabe K., Zhou Y. (2024). Electrical control
and transport of tightly bound interlayer excitons in a mose_2_/hBN/mose_2_ heterostructure. Phys.
Rev. Lett..

[6] Baek H., Brotons-Gisbert M., Koong Z. X., Campbell A., Rambach M., Watanabe K., Taniguchi T., Gerardot B. D. (2020). Highly energy-tunable
quantum light from moiré-trapped excitons. Science Advances.

[7] Wang G., Chernikov A., Glazov M. M., Heinz T. F., Marie X., Amand T., Urbaszek B. (2018). Colloquium: Excitons in atomically
thin transition metal dichalcogenides. Rev.
Mod. Phys..

[8] Unuchek D., Ciarrocchi A., Avsar A., Sun Z., Watanabe K., Taniguchi T., Kis A. (2019). Valley-polarized exciton currents
in a van der waals heterostructure. Nat. Nanotechnol..

[9] Policht V. R., Russo M., Liu F., Trovatello C., Maiuri M., Bai Y., Zhu X., Dal Conte S., Cerullo G. (2021). Dissecting Interlayer Hole and Electron
Transfer in
Transition Metal Dichalcogenide Heterostructures via Two-Dimensional
Electronic Spectroscopy. Nano Lett..

[10] Rodriguez A., Kalbáč M., Frank O. (2021). Strong localization effects in the
photoluminescence of transition metal dichalcogenide heterobilayers. 2D Materials.

[11] Garrity O., Rodriguez A., Mueller N. S., Frank O., Kusch P. (2022). Probing the
local dielectric function of WS2 on an Au substrate by near field
optical microscopy operating in the visible spectral range. Appl. Surf. Sci..

[12] Govyadinov A. A., Amenabar I., Huth F., Carney P. S., Hillenbrand R. (2013). Quantitative
measurement of local infrared absorption and dielectric function with
tip-enhanced near-field microscopy. J. Phys.
Chem. Lett..

[13] Scuri G., Zhou Y., High A. A., Wild D. S., Shu C., De Greve K., Jauregui L. A., Taniguchi T., Watanabe K., Kim P., Lukin M. D., Park H. (2018). Large excitonic
reflectivity of monolayer mose_2_ encapsulated in hexagonal
boron nitride. Phys. Rev. Lett..

[14] Glazov M. M., Amand T., Marie X., Lagarde D., Bouet L., Urbaszek B. (2014). Exciton fine structure
and spin decoherence in monolayers
of transition metal dichalcogenides. Phys. Rev.
B.

[15] Hauer B., Engelhardt A. P., Taubner T. (2012). Quasi-analytical model for scattering
infrared near-field microscopy on layered systems. Opt. Express.

[16] Castellanos-Gomez A., Buscema M., Molenaar R., Singh V., Janssen L., Van Der Zant H. S., Steele G. A. (2014). Deterministic transfer of two-dimensional
materials by all-dry viscoelastic stamping. 2D Materials.

[17] Nayak P. K., Horbatenko Y., Ahn S., Kim G., Lee J. U., Ma K. Y., Jang A. R., Lim H., Kim D., Ryu S., Cheong H., Park N., Shin H. S. (2017). Probing Evolution
of Twist-Angle-Dependent Interlayer Excitons in MoSe2/WSe2 van der
Waals Heterostructures. ACS Nano.

[18] Jain A., Bharadwaj P., Heeg S., Parzefall M., Taniguchi T., Watanabe K., Novotny L. (2018). Minimizing residues
and strain in 2d materials transferred from pdms. Nanotechnology.

[19] Ghiami A., Fiadziushkin H., Sun T., Tang S., Wang Y., Mayer E., Schneider J. M., Piacentini A., Lemme M. C., Heuken M., Kalisch H., Vescan A. (2025). Improved reliability
of 2d-tmdc dry transfer via pmma and target substrate treatments. ACS Applied Electronic Materials.

[20] Keilmann F., Hillenbrand R. (2004). Near-field
microscopy by elastic light scattering from
a tip. Philosophical Transactions of the Royal Society of London.
Series A: Mathematical. Physical and Engineering
Sciences.

[21] Kusch P., Arcos Pareja J. A., Tsarapkin A., Deinhart V., Harbauer K., Höflich K., Reich S. (2024). Double tips for in-plane polarized
near-field microscopy and spectroscopy. Nano
Lett..

[22] Ocelic N., Huber A., Hillenbrand R. (2006). Pseudoheterodyne detection for background-free
near-field spectroscopy. Appl. Phys. Lett..

[23] Mester L., Govyadinov A. A., Hillenbrand R. (2022). High-fidelity nano-FTIR spectroscopy
by on-pixel normalization of signal harmonics. Nanophotonics.

[24] Malitson I. H. (1965). Interspecimen
comparison of the refractive index of fused silica*,†. J. Opt. Soc. Am..

[25] Mueller N. S., Vieira B. G. M., Schulz F., Kusch P., Oddone V., Barros E. B., Lange H., Reich S. (2018). Dark Interlayer Plasmons
in Colloidal Gold Nanoparticle Bi- and Few-Layers. ACS Photonics.

[26] Juergensen S., Kessens M., Berrezueta-Palacios C., Severin N., Ifland S., Rabe J. P., Mueller N. S., Reich S. (2023). Collective States in
Molecular Monolayers on 2D Materials. ACS Nano.

[27] Zhang S., Li B., Chen X., Ruta F. L., Shao Y., Sternbach A. J., McLeod A. S., Sun Z., Xiong L., Moore S. L., Xu X., Wu W., Shabani S., Zhou L., Wang Z., Mooshammer F., Ray E., Wilson N., Schuck P. J., Dean C. R., Pasupathy A. N., Lipson M., Xu X., Zhu X., Millis A. J., Liu M., Hone J. C., Basov D. N. (2022). Nano-spectroscopy
of excitons in atomically thin transition metal dichalcogenides. Nat. Commun..

[28] Cvitkovic A., Ocelic N., Hillenbrand R. (2007). Analytical
model for quantitative
prediction of material contrasts in scattering-type near-field optical
microscopy. Opt. Express.

[29] Chen X., Ren R., Liu M. (2021). Validity of machine learning in the quantitative analysis
of complex Scanning Near-Field Optical Microscopy Signals Using Simulated
Data. Physical Review Applied.

[30] Li W., Birdwell A. G., Amani M., Burke R. A., Ling X., Lee Y. H., Liang X., Peng L., Richter C. A., Kong J., Gundlach D. J., Nguyen N. V. (2014). Broadband optical
properties of large-area monolayer CVD molybdenum disulfide. Physical Review B - Condensed Matter and Materials Physics.

[31] Casses L. N., Zhou B., Lin Q., Tan A., Bendixen-Fernex
de Mongex D.-P., Kaltenecker K. J., Xiao S., Wubs M., Stenger N. (2024). Full quantitative near-field characterization of strongly
coupled exciton–plasmon polaritons in thin-layered wse2 on
a monocrystalline gold platelet. ACS Photonics.

[32] Santos E. J. G., Kaxiras E. (2013). Electrically Driven
Tuning of the Dielectric Constant
in MoS2 Layers. ACS Nano.

[33] Griffiths, D. J. Introduction to Electrodynamics; Pearson Higher Education, 2014. Google-Books-ID: J9ygBwAAQBAJ.

[34] Sumi H., Kayanuma Y. (1993). Is the Lorentz model applicable to optical absorption
by excitons?. Solid State Commun..

[35] Moody G., Schaibley J., Xu X. (2016). Exciton dynamics in monolayer transition
metal dichalcogenides. J. Opt. Soc. Am. B.

[36] Garrity O., Brumme T., Bergmann A., Korn T., Kusch P., Reich S. (2024). Interlayer Exciton–Phonon
Coupling in MoSe2/WSe2 Heterostructures. Nano
Lett..

[37] Roy S., Sharbirin A. S., Lee Y., Kim W. B., Kim T. S., Cho K., Kang K., Jung H. S., Kim J. (2020). Measurement of Quantum
Yields of Monolayer TMDs Using Dye-Dispersed PMMA Thin Films. Nanomaterials.

[38] Kim H., Ahn G. H., Cho J., Amani M., Mastandrea J. P., Groschner C. K., Lien D.-H., Zhao Y., Ager J. W., Scott M. C., Chrzan D. C., Javey A. (2019). Synthetic WSe2 monolayers
with high photoluminescence quantum yield. Science
Advances.

[39] Rivera P., Schaibley J. R., Jones A. M., Ross J. S., Wu S., Aivazian G., Klement P., Seyler K., Clark G., Ghimire N. J., Yan J., Mandrus D. G., Yao W., Xu X. (2015). Observation of long-lived interlayer excitons in monolayer MoSe2–WSe2
heterostructures. Nat. Commun..

[40] Rigosi A. F., Hill H. M., Li Y., Chernikov A., Heinz T. F. (2015). Probing Interlayer Interactions in
Transition Metal
Dichalcogenide Heterostructures by Optical Spectroscopy: MoS2/WS2
and MoSe2/WSe2. Nano Lett..

[41] Miller B., Steinhoff A., Pano B., Klein J., Jahnke F., Holleitner A., Wurstbauer U. (2017). Long-Lived Direct and Indirect Interlayer
Excitons in van der Waals Heterostructures. Nano Lett..

[42] Sigger F., Lambers H., Nisi K., Klein J., Saigal N., Holleitner A. W., Wurstbauer U. (2022). Spectroscopic imaging ellipsometry
of two-dimensional TMDC heterostructures. Appl.
Phys. Lett..

[43] Torun E., Miranda H. P., Molina-Sánchez A., Wirtz L. (2018). Interlayer
and intralayer excitons in MoS2/WS2 and MoSe2/WSe2 heterobilayers. Phys. Rev. B.

[44] Meier S., Zhumagulov Y., Dietl M., Parzefall P., Kempf M., Holler J., Nagler P., Faria Junior P. E., Fabian J., Korn T., Schuller C. (2023). Emergent trion-phonon
coupling in atomically reconstructed mose2-wse2 heterobilayers. Physical Review Research.

