# Supplementary material for: Characterizing Interlayer Excitons by Spectral Signature in Scattering Visible Near-Field Microscopy
Source: J Phys Chem Lett. 2025 Jun 30;16(27):6960–7. doi: 10.1021/acs.jpclett.5c01052 (PMC12257585; doi:10.1021/acs.jpclett.5c01052)
Supplement: Supplementary file 1 [file jz5c01052_si_001.pdf]

Supplementary Material: Characterising Interlayer Excitons by Spectral Signature in Scattering Visible Near-Field Microscopy

Oisín Garrity*,[1] Iris Niehues,[2] Annika Bergmann-Iwe,[3] Anna Wróblewska,[4] Luka Pirker,[5] Adeel Bukhari,[6,5] Gregor Hlawacek,[7] Tobias Korn,[3] Otakar Frank,[5] and Patryk Kusch[1]

[1]*Department of Physics, Freie Universität Berlin, Arnimallee 14, D-14195 Berlin, Germany*

[2]*Institute of Physics, University of Münster, 48149, Münster, Germany*

[3]*Institute of Physics, Universität Rostock, 18059 Rostock, Germany*

[4]*Faculty of Physics, Warsaw University of Technology, Koszykowa 75, 00-662, Warsaw, Poland*

[5]*J. Heyrovský Institute of Physical Chemistry of the CAS, Dolejškova 2155/3, 182 00 Prague 8, Czech Republic*

[6]*Faculty of Mathematics and Physics, Charles University, Ke Karlovu 3, 12116, Prague, Czech Republic*

[7]*Helmholtz-Zentrum Dresden–Rossendorf, Institut für Ionenstrahlphysik und Materialforschung, D-01328 Dresden, Germany*

* Corresponding author: `o.garrity@fu-berlin.de`

THEORETICAL MODEL FOR NEAR-FIELD RESPONSE

To theoretically model the near-field response, we use the finite dipole model for multi-layer systems [1, 2]. Considering a metallic tip exposed to an incident electric field E_{in}, a dipole moment is induced in the tip, given by

$$p_{\text{total}} = \alpha_{\text{eff}} E_{\text{in}} \, , \tag{S1}$$

where the effective polarizability α_{eff} quantifies the response of the dipole moment to external fields, including modifications by the sample.

Bringing the tip near a dielectric interface (e.g., MoS_2, $MoSe_2/WSe_2$ heterostructure) modifies the dipole moment due to charge redistribution within the sample, influencing α_{eff}. To determine α_{eff}, we first establish the relevant approximations.

Since the tip-sample distance (H=10 − 30 nm) is much smaller than the wavelength of light ($\lambda = 600 - 800$ nm), the quasi-static approximation applies. This assumes that electric fields behave as if they are static, interactions are dominated by evanescent fields, and time-dependent/magnetic field effects become negligible. Under this approximation, the potential satisfies Laplace's equation:

$$\nabla^2 \phi = 0 \, . \tag{S2}$$

The tip-sample system is modeled using the method of images, where the dielectric function of the sample modifies the charge distribution of the tip.

Induced Charge Distribution

The electric field E_{in} incident on the metallic spheroid tip induces a dipolar charge distribution at its apex. The dielectric response of the sample alters this charge distribution, which is modeled using image charges. The primary induced dipole is given by

$$p_0 \approx 2 W_0 Q_0 \, , \tag{S3}$$

where $\pm Q_0$ are point charges located at distances

$$W_0 = \frac{1.31 RL}{L + 2R} \, . \tag{S4}$$

The distances denoted W specify the positions of induced charges along the spheroid's axis. These positions emerge from solving Laplace's equation inside a toroidal spheroidal volume and ensuring continuity of the potential at the surface.

Due to the distance of the point charge $-Q_0$ from the sample surface, its direct contribution to the near-field interaction is negligible.

Momentum-Space Representation and Boundary Conditions

Because the tip's near-field interaction extends over a finite region, the response must be described as a sum over all in-plane momentum components rather than a single spatial coordinate. This formulation captures the full effect of spatially varying near-field interactions. The total potential near the tip is expressed as:

$$\Phi_{\text{tot}}(z) = \int_0^\infty \hat{\phi}(q) e^{-qz} dq \,, \tag{S5}$$

where q is the in-plane wavevector, and $\hat{\phi}(q)$ is the momentum-space representation of the potential.

To determine how the sample modifies the near-field interaction, we impose electrostatic boundary conditions at the interface. Specifically:

$$E_{||}^{\text{tip}} = E_{||}^{\text{sample}} \,, \tag{S6}$$

which ensures continuity of the in-plane electric field, and

$$D_{\perp}^{\text{tip}} = D_{\perp}^{\text{sample}} \,, \tag{S7}$$

which ensures continuity of the normal displacement field.

These conditions prevent artificial surface charge accumulation while enforcing a momentum-dependent dielectric response $\epsilon(q)$. This modifies the sample's potential via

$$\phi_{\text{sample}}(q) = \beta(q)\, \phi_{\text{tip}} \,, \tag{S8}$$

where

$$\beta(q) = \frac{\epsilon_1\, k_{z2} - \epsilon_2\, k_{z1}}{\epsilon_1\, k_{z2} + \epsilon_2\, k_{z1}} \,. \tag{S9}$$

Here, $\beta(q)$ is the Fresnel reflection coefficient, which determines how much of the sample's induced field is reflected back and modifies the tip's effective dipole.

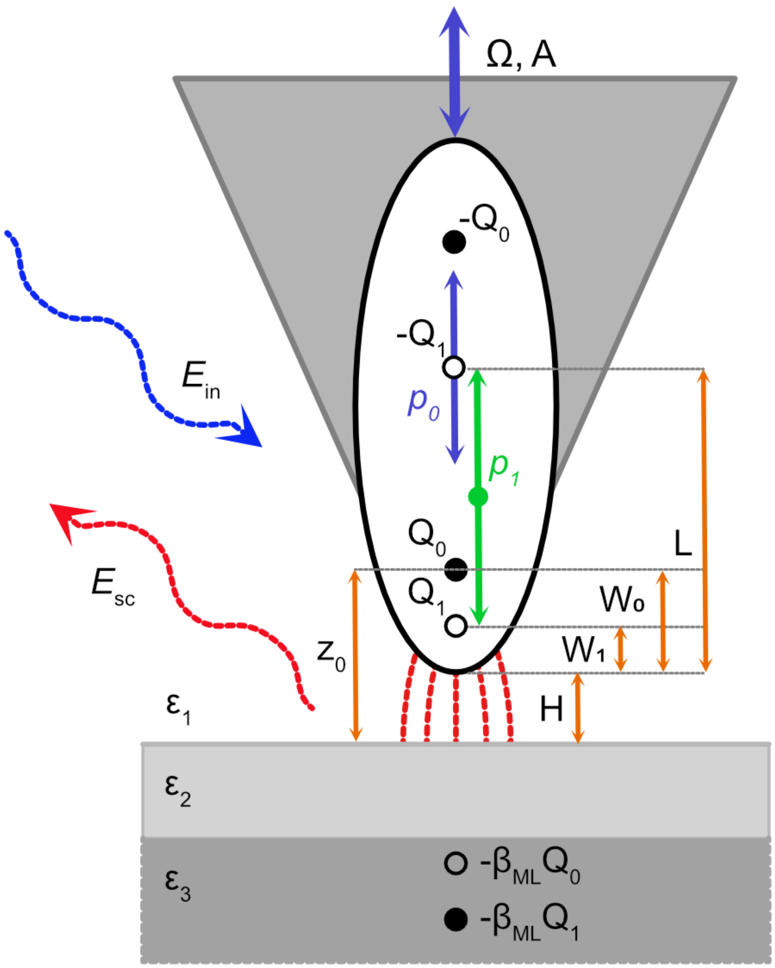

FIG. S1. The finite dipole model (FDM) for near-field interactions. The tip is approximated as a perfectly conducting prolate spheroid, polarized by the incident field E_{in}. The field at the apex is represented by two point charges, $\pm Q_0$, forming a dipole $p_0 \approx 2LQ_0$, where $2L$ is the spheroid length. The near-field interaction is modeled using the method of images, where the sample induces mirror charges $-\beta_{ij}Q_0$, with β_{ij} as the Fresnel reflection coefficient. The induced near-field creates a secondary dipole, $p_1 \approx Q_1L$, modifying the effective polarizability α_{eff}. The effective polarizability depends on the tip geometry, specifically the tip-sample distance H and apex-charge distances W_1 and W_2. The tip oscillates at a tapping frequency Ω with an amplitude A. Figure adapted from [3].

Recursive Induction of Mirror Charges

The tip dipole creates an image charge in the sample:

$$p_0 = -\beta(q) Q_0 \,, \tag{S10}$$

where the minus sign indicates an opposite dipole to that of the tip. This image charge, in turn, induces a secondary dipole in the tip:

$$p_1 = -\beta(q) p_0 \,. \tag{S11}$$

Repeating this process leads to an infinite sequence of reflections:

$$p_{\text{total}} = p_0 + p_1 + p_2 + p_3 + \dots \tag{S12}$$

where

$$p_n = p_0 \left(-\beta(q)\right)^n . \tag{S13}$$

This forms an infinite geometric series:

$$p_{\text{total}} = p_0 \sum_{n=0}^{\infty} (-\beta(q))^n \,. \tag{S14}$$

For $|\beta(q)| < 1$, and using $p_0 \approx 2Q_0 W_0$, this sum converges to:

$$p_{\text{total}} = \frac{2Q_0 W_0}{1+\beta(q)} \,. \tag{S15}$$

Coupling Weight Functions and Final Effective Polarizability

Extending from discrete point charges to a continuous charge distribution along the spheroid, the induced charge strength is calculated by solving Laplace's equation. The mirror charges introduce screening effects that modify the tip's total dipole moment. These effects are accounted for using coupling weight functions:

$$f_0(q) = \ln\left(\frac{W_0}{W_1}\right), \quad f_1(q) = \ln\left(\frac{W_1}{H}\right), \tag{S16}$$

where H is the tip-sample distance. These functions describe how charge redistributes along the tip and determine the effective mirror charge response.

Finally, incorporating these into Eq. (S15), the effective polarizability is:

$$\alpha_{\text{eff}} \propto \frac{\beta f_0}{2(1-\beta f_1)}. \tag{S17}$$

which includes the tip-induced dipole modified by the sample response $\beta(q)$, and the weight functions $f_0(q)$ and $f_1(q)$, which account for the redistribution of induced charge along the tip's surface. In the case of a semi-infinite dielectric sample, this formulation describes the tip's dipole response in the presence of a single dielectric interface, modeled via the method of images. However, for multilayered systems, a more generalized reflection coefficient is required to account for multiple interfaces, necessitating an extension of the model.

FINITE DIPOLE MODEL FOR MULTILAYER SYSTEMS

The samples studied in this work consist of **three-layered dielectric interfaces**, specifically **MoS_2 or $MoSe_2/WSe_2$ heterostructures on a Si/SiO_2 substrate**. While the finite dipole model derived in the previous section accurately describes a **semi-infinite dielectric interface**, it must be extended to account for **multiple interfaces**. This extension requires three key modifications:

1. Generalizing the **reflection coefficient** $\beta(q)$ to incorporate the layered dielectric structure of **MoS_2, WSe_2, and the underlying SiO_2 substrate**.
2. **Reformulating the image charge distribution** to include contributions from all interfaces, rather than a single semi-infinite medium.
3. Extending the **effective polarizability** α_{eff} to capture multilayer influences, ensuring the near-field response correctly accounts for each interface's dielectric response.

To begin, for a single interface with layer 1 (ϵ_1) and layer 2 (ϵ_2), the standard Fresnel reflection coefficient in momentum space is

$$\beta(q) = \frac{\epsilon_1 \, k_{z2} - \epsilon_2 \, k_{z1}}{\epsilon_1 \, k_{z2} + \epsilon_2 \, k_{z1}} \, ,$$

where $k_{zi} = \sqrt{\epsilon_i \frac{\omega^2}{c^2} - q^2}$ is the out-of-plane wavevector component. Now, instead of a semi-infinite second layer, an additional layer (MoS_2 or $MoSe_2/WSe_2$) is added between air and the semi-infinite layer SiO_2 with a thickness d_2. Extra layers come with additional reflections so

we must stack multiple reflection coefficients recursively. The preferred notation in near-field optics for an effective reflection coefficient is β_{ML}, similar to the Fresnel reflection coefficient r_{ij} in optics due to its recursive nature:

$$\beta_{\mathrm{ML}} = \frac{\epsilon_j\, k_{zi} - \epsilon_i\, k_{zj}}{\epsilon_j\, k_{zi} + \epsilon_i\, k_{zj}}\,,$$

this form tracks reflections across multiple interfaces thus in order to find the cumulative reflection coefficient for 3 layers (two interfaces) you add them iteratively. The reflection coefficient between layers 1 and 2:

$$\beta_{12} = \frac{\epsilon_2\, k_{z1} - \epsilon_1\, k_{z2}}{\epsilon_2\, k_{z1} + \epsilon_1\, k_{z2}}\,.$$

Similarly, for the interface between layer 2 and 3:

$$\beta_{23} = \frac{\epsilon_3\, k_{z2} - \epsilon_2\, k_{z3}}{\epsilon_3\, k_{z2} + \epsilon_2\, k_{z3}}\,.$$

In a three-layer system, light partially transmits through layer 2 and reflects back and forth until transmitting through to layer 3. Every time the light reflects inside layer 2, it picks up a phase shift e^{-2qd_2}, where q is the in-plane momentum, due to its finite thickness d_2. The phase factor accounts for the exponential decay of evanescent waves inside the layer. To fully describe multiple reflections in a layered system, we iteratively sum all contributions, accounting for the phase shift acquired during each round trip inside layer 2. The first reflection is described by β_{12}, the next reflection after a round trip in layer 2 is described by $\beta_{23}\, e^{-2\,q\,d_2}$, and with another double journey in the third layer described by $\beta_{12}\beta_{23}e^{-4\,q\,d_2}$ they can be summed:

$$\beta_{\mathrm{ML}} = \beta_{12} + \beta_{23}e^{-2\,q\,d_2} + \beta_{12}\beta_{23}e^{-4\,q\,d_2} + \beta_{12}\beta_{23}^2e^{-6\,q\,d_2} + ...$$

This forms a geometric series:

$$\sum_{n=0}^{\infty} A^n = \frac{1}{1-A}\,, \quad \text{for } |A| < 1\,,$$

where, if we identify $A = \beta_{12}\beta_{23}e^{-2\,q\,d_2}$ and $|\beta_{12}\beta_{23}e^{-2\,q\,d_2}| < 1$, this leads to:

$$\beta_{\mathrm{ML}} = \beta_{12} + \frac{\beta_{23}e^{-2\,q\,d_2}}{1 - \beta_{12}\beta_{23}e^{-2\,q\,d_2}}\,, \tag{S18}$$

which is the recursive reflection coefficient used in this work and the explicit form found in the Mathematica code used for fitting. Physically, β_{ML} encapsulates the full dielectric response of

the layered sample, including all multiple scattering effects within intermediate layers. The recursive form of the reflection coefficient derived here via the geometric series approach is mathematically equivalent to the result obtained using the transfer matrix formalism, where the total reflection is iteratively constructed from individual interface contributions and phase factors.

Next is to modify the effective image charge and strength to incorporate reflections from multiple interfaces. The total reflected potential $\phi(z)$ can be described using the cumulative reflection coefficient β_{ML} and integrated over all in-plane momenta q:

$$\Phi(z) = \int_0^\infty \beta_{\mathrm{ML}}(q) e^{-2q\,z} e^{-q\,z} dq \tag{S19}$$

with $e^{-2q\,z}$ accounting for the initial image charge response, and $e^{-q\,z}$ ensuring the exponential decay of near-field interactions.

To find the effective dipole moment, we must define the image charge strength and position in terms of the reflected potential, Eq. (S19). Thus to approximate the potential response of the sample to an image charge $-Q_0 = -\beta X Q$ located a distance X below the interface leading to boundary conditions at $z = 0$:

$$\Phi\Big|_{z=0} = \frac{-\beta X}{z_0 + X}$$

which ensures potential continuity at the interface, in addition to:

$$\Phi' = \frac{\partial \Phi}{\partial z}\Big|_{z=0} = -\frac{\beta X}{(z_0 + X)^2}$$

that ensures that the electric field discontinuity follows from the sample's permittivity. From these two conditions, solving for the image charge strength βX and the image charge position X:

$$\beta X = -\frac{\Phi^2}{\Phi'}\Big|_{z=0}, \quad X = \frac{\Phi}{\Phi'}\Big|_{z=0} - z_0\,, \tag{S20}$$

Finally to determine the effective polarisibility, the steps are the same as the semi-infinite case where the total induced dipole is used to relate the effective polarisibilty to the total reflected potential. To obtain the induced dipole moment, we differentiate $\Phi(q)$ with respect to z:

$$E_{\mathrm{ind}} = \frac{\partial \Phi(z)}{\partial z}\Big|_{z=0}.$$

Since the dipole moment is proportional to the induced electric field,

$$p_{\mathrm{total}} \propto E_{\mathrm{ind}} \propto \int_0^\infty \beta_{\mathrm{ML}}(q) e^{-2q\,z} e^{-q\,z} dq\Big|_{z=0}.$$

Since the total dipole moment p_{total} depends on the reflected potential Φ, and α_{eff} is defined via $p_{\text{total}} = \alpha_{\text{eff}} E_{\text{in}}$, we arrive at the final form by integrating over all wavevectors.:

$$\alpha_{\text{eff}} \propto \frac{\beta_{\text{ML}}(q) f_0}{2(1 - \beta_{\text{ML}} f_1)} \,. \tag{S21}$$

This is the form of the equation that is in the Mathematica code to fit the spectral signatures of MoS_2 and $MoS2_2/WSe_2$ heterostructures from data taken from s-SNOM images. It is a function of the cumulative reflection coefficient β_{ML} and of the tip-sample height H (hidden in the coupling weight functions $f_{0,1}$). In practical s-SNOM measurements, the AFM tip oscillates at a frequency Ω with amplitude A, leading to a time-dependent tip-sample distance:

$$H(t) = H_0 + A \cos \Omega t \,. \tag{S22}$$

This oscillation modulates the near-field interaction, and its influence is extracted through higher harmonic demodulation. In the following section, we present raw near-field amplitude images and discuss the signal processing steps required to isolate the material response.

VALIDATING THE MULTILAYER FDM ON PRISTINE 1L-MOS_2/HBN

To benchmark the multilayer FDM model, we took s-SNOM measurement of pristine 1L-MoS_2 on 10 nm of hBN through its B-exciton resonance, Fig. S2 (a). The images were normalised by the Si substrate and by dividing by the previous harmonic. In the 4th harmonic amplitude images, Fig. S2 (b) - 1.94 eV and Fig. S2 (c) - 2.14 eV, there is a constrast shift evident in the MoS_2. Taking an average of the near-field contrast, averaging area indicated by the red region in Fig. S2 (a), and plotting it against excitation energy yields a Lorentzian line shape. Using the multilayer FDM model, the data was then fitted with a Lorentzian oscillator, yielding a resonance energy of 1.98 eV and a Γ_B of 30 meV. These values have excellent agreement with the PL spectra and literature values, Table S1.

TABLE S1. Comparison of B-exciton parameters for pristine 1L-MoS_2 from photoluminescence (PL), near-field model fit (FDM), and selected literature values at room temperature.

Parameter	PL	FDM Fit	Literature
Peak energy (eV)	1.878	1.980	1.97–2.01 [4]
FWHM Γ (meV)	48	30	30–50 [4]

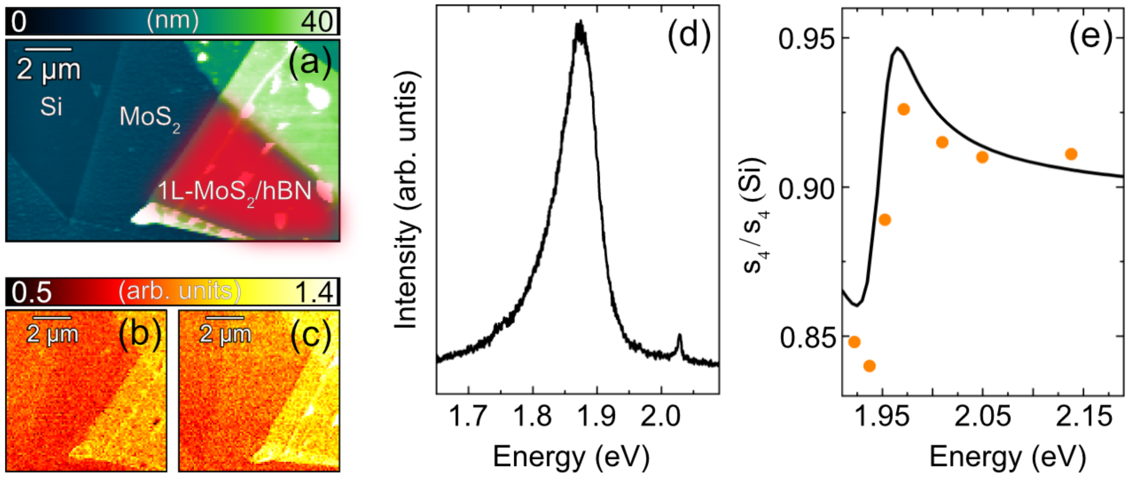

FIG. S2. 1L-MoS_2/hBN (10 nm) showing the B exciton resonance at 1.98 eV. (a) AFM image of the sample, with the monolayer region on hBN indicated. (b, c) Near-field amplitude images recorded at 1.94 eV and 2.14 eV, respectively. (d) PL Spectra from the 1L-MoS_2 flake. (e) Spectral response extracted by averaging over the red-shaded region in (a), normalized to the Si substrate. The solid line is a fit using the multilayer finite dipole model.

4L-MOS$_2$ IRRADIATION

To study the effect of defects on the optical properties of the 4L-MoS$_2$, and to see if the s-SNOM are sensitive to these changes, we irradiated the flake with a He+ ion beam with a diameter of 0.5 nm with an ion energy of 7.5 keV. Before irradiation, the 4L-MoS$_2$ flake exhibited strong PL response, with the B-exciton present at 1.98 eV with a FWHM of 30 meV, Fig. S2(a). Upon irradiation, it's clear from the PL response, Fig. S2(b), that significant broadening was introduced. The peak energy is lost in the noise and but using the peak positions from the pristine PL spectra, a plausible Voight profile fit was obtained revealing a FWHM of 132 meV. While the fitted FWHM (Γ_B) is lower at 50 meV, this is because the FWHM in PL incorporates more broadening mechanisms than near-field probing does. A table containing the peak energies and FWHM of irradiated and non-irradiated MoS$_2$ can be found in Table S2. To study the effect of disorder on the optical response of 4L-MoS$_2$, and to assess the sensitivity of s-SNOM to such changes, the flake was irradiated with a He$^+$ ion beam (0.5 nm diameter, 7.5 keV ion energy). Prior to irradiation, the flake

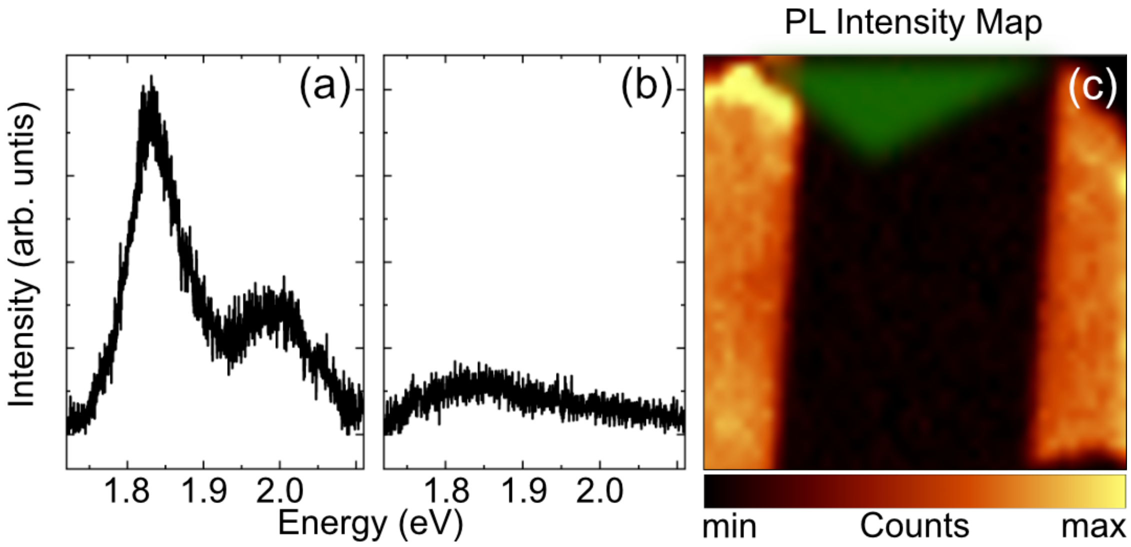

FIG. S3. PL spectra from pristine (a) and irradiated (b) regions of 4L-MoS$_2$, with the corresponding PL intensity map shown in (c). The irradiated region exhibits suppressed emission and broadening of both excitonic peaks. Voigt profile fits (not shown) yield A and B exciton linewidths of 293 meV and 132 meV, respectively. The B exciton remains observable near 2.02 eV, consistent with the resonance identified in the s-SNOM analysis. The green-shaded area in (c) indicates the position of the 4L-MoS$_2$ flake.

TABLE S2. Comparison of B-exciton peak energies and linewidths (FWHM Γ) for 4L-MoS_2 under pristine and irradiated conditions, extracted from photoluminescence (PL) and near-field FDM model fits. The PL results reveal pronounced broadening in the irradiated region, consistent with disorder-induced damping. FDM model fits yield reduced linewidths, as expected for near-field measurements. Literature values correspond to reported multilayer B-exciton features at room temperature [5].

**4L-MoS_2**	**Peak Energy (eV)**	**FWHM Γ (meV)**
Irradiated PL	2.02	132
Irradiated FDM fit	2.01	50
Pristine PL	1.98	30
Literature (room temp)	2.01	30 [5]

exhibited strong photoluminescence (PL), with the B-exciton centered at 1.98 eV and a full-width at half-maximum (FWHM) of 30 meV, as shown in Fig. S2(a). After irradiation, the PL signal was strongly quenched, as seen in Fig. S2(b), and both A- and B-exciton peaks showed significant broadening. While the B-exciton peak position is not clearly resolved in the irradiated spectrum, a Voigt profile fit using the pristine peak positions yielded a FWHM of 132 meV for the B-exciton. In comparison, the near-field FDM model applied to the same region produced a fitted linewidth $\Gamma_B = 50\,\text{meV}$, which is narrower as expected, since PL captures additional broadening mechanisms not present in the near-field response. A summary of the extracted peak energies and linewidths is presented in Table S2.

DEMODULATION AND REFLECTION SUPPRESSION IN S-SNOM

The s-SNOM near-field signal is mixed with strong far-field contributions. To uncouple the unwanted far-field contributions from the pure near-field signal, higher-harmonic demodulation is used. With the tip oscillating at a frequency Ω and an amplitude A, Eq. (S22), the signal is analysed at multiple harmonics s_n. This oscillation modulates the scattered field but the near-field and far field components of the signal respond differently. The near-field response is highly non-linear due to the strong dependence of the interaction on the tip-sample distance, Eq. (S22). This leads to the near-field signal containing higher harmonics of the oscillation frequency (i.e., s_n at $n\Omega$). In contrast, the far-field response is largely linear resulting in most of it contributing to the fundamental harmonic rather than higher harmonics. Thus demodulating at $n > 1$ selectively enhances the near-field signal over the far-field signal [6].

To accurately extract both amplitude and phase information, a pseudo-heterodyne detection scheme is employed. In this method, phase modulation is introduced in the reference arm of a Michelson interferometer by oscillating the reference mirror. The recorded interference pattern as a function of mirror displacement allows Fourier-based extraction of both amplitude and phase components of the near-field signal. This method not only improves noise suppression but also enables simultaneous retrieval of amplitude and phase images. For more on this topic, please refer to Ocelic *et.al.* [6].

To suppress spurious reflections arising from edges, cantilever interactions, and inhomogeneities, we applied harmonic background correction. Specifically, the $n = 4$ amplitude images were normalized by their $n = 3$ counterparts, and the $n = 4$ phase images were corrected by subtracting the corresponding $n = 3$ phase images. This approach helps isolate the intrinsic near-field response by reducing residual multiple reflection artifacts.

All these background suppression techniques ensure that the image is dependent solely on the near-field interaction, and we can extract spectral signatures of excitons from the s-SNOM images due to the near-field response being a function of tip-sample height and the dielectric function (through the reflection coefficient). This is what the main manuscript covers in detail. However, below features examples of the s-SNOM images used in the manuscript.

To investigate the spectral response of MoS_2 and $MoSe_2/WSe_2$, s-SNOM images were ac-

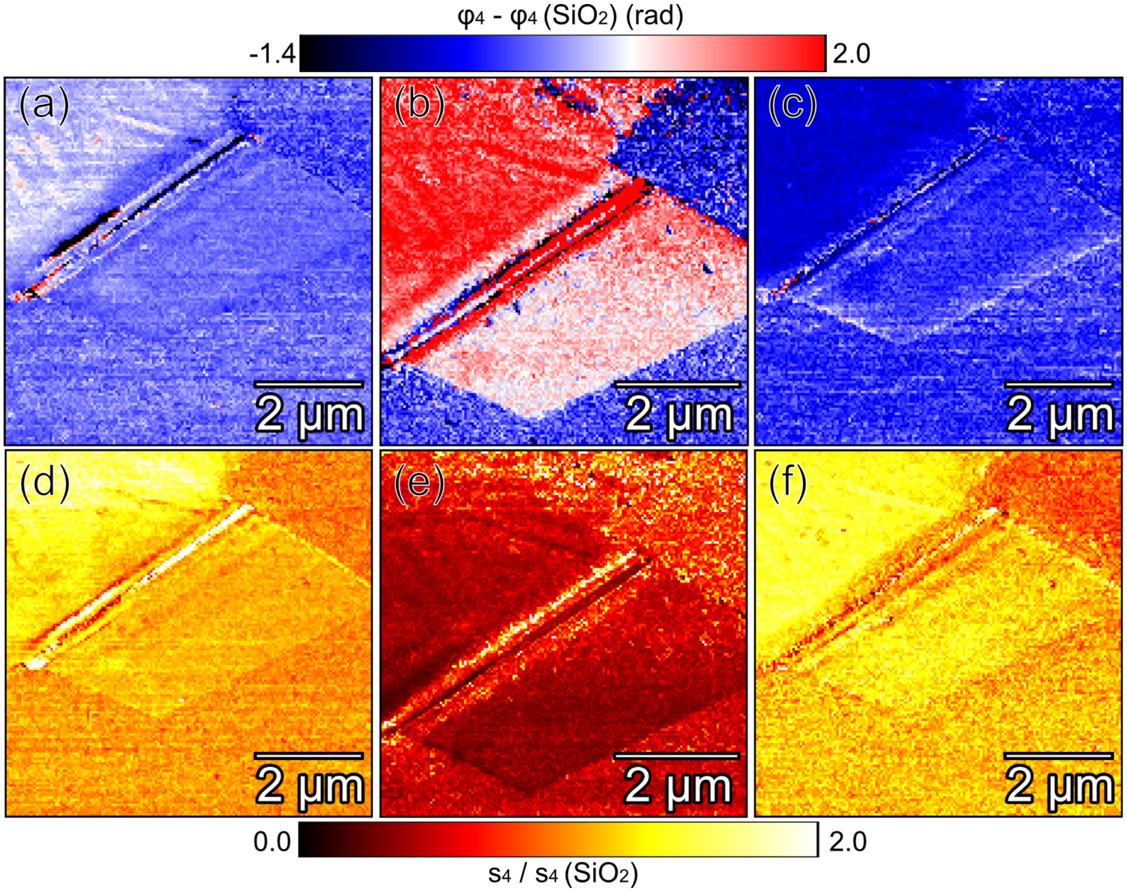

FIG. S4. Near-field optical images of four-layer MoS_2 (4L-MoS_2) acquired using s-SNOM at three different excitation energies: 1.97 eV, 2.00 eV, and 2.14 eV. (a-c) Phase images, referenced to SiO_2. (d-f) Amplitude images, normalized to SiO_2. Higher-order reflection artifacts were removed by division (amplitude) and subtraction (phase). The contrast change at 2.00 eV corresponds to the XB^M exciton resonance, as discussed in the main text.

quired at multiple excitation energies, Fig. S4 and Fig. S5, focusing on excitonic resonances. These images provide spatially resolved information on the near-field optical contrast, which is sensitive to the local dielectric function. Below, we present representative s-SNOM images for both MoS_2 and the $MoSe_2/WSe_2$ heterostructure. The images were taken at 1.97, 2.00, and 2.14 eV. In Fig. S4, the near-field optical contrast of 4L-MoS_2 is shown across the XBM exciton resonance at 1.97 eV, 2.00 eV, and 2.14 eV. At 2.00 eV, a clear increase in near-field contrast is observed, highlighting the excitonic resonance. This enhanced contrast, visible in both phase and amplitude images, results from the strong dielectric response of the XBM exciton in MoS_2. To quantify this effect, the average near-field contrast was extracted from

the MoS_2 region and plotted as a function of excitation energy in the main manuscript.

Fig. S5 presents s-SNOM images of the $MoSe_2/WSe_2$ heterostructure, emphasizing the response of the interlayer exciton (IX). The IX active region, highlighted by a dashed circle, exhibits strong near-field contrast at 1.35 eV, corresponding to the expected interlayer transition energy. To further confirm the local dielectric response, we examined the contrast as a function of demodulation order, leveraging the higher harmonics to enhance near-field sensitivity. Panels (a-c) present optical phase images at $n = 3$ demodulation, while panels (d-f) show optical amplitude images at $n = 4$ demodulation, taken at 1.34 eV, 1.35 eV, 1.37 eV. The use of higher harmonic demodulation ($n > 1$) ensures that the primary contribution is from the near-field response, with additional noise suppression techniques applied. The spatial distribution of the IX signal is observed as a function of demodulation order, confirming the sensitivity of the technique to local dielectric variations. The evolution of IX contrast across different harmonics confirms the sensitivity of s-SNOM to local dielectric variations, demonstrating its ability to resolve excitonic interactions at the nanoscale.

The area for the 4L-MoS_2 where the average s-SNOM constrast was taken is within the boundary of the slab clearly seen in Fig. S4 (also indicated in the main text, Fig. 2). The averaged area taken for the IX active HT indicated by the black dashed circle in Fig. S5(a) (also indicated in Fig. 3 and Fig. 4 of the main text).

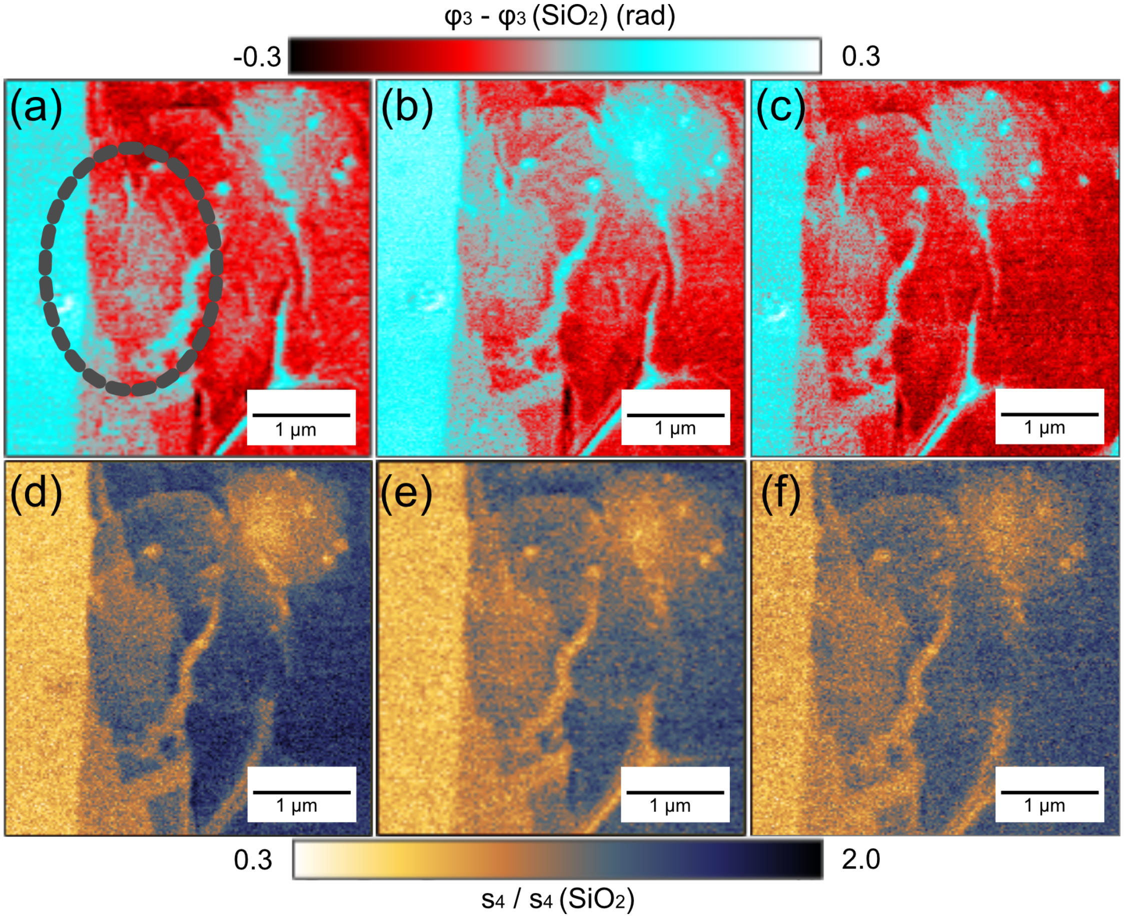

FIG. S5. Near-field optical images of the $MoSe_2/WSe_2$ heterostructure (HT) acquired using s-SNOM at 1.34 eV, 1.35 eV, and 1.37 eV. (a-c) Phase images at third-harmonic demodulation ($n = 3$). (d-f) Amplitude images at fourth-harmonic demodulation ($n = 4$). The interlayer exciton (IX) active region is indicated by a dashed circle. Normalization to SiO_2 and artifact removal were applied as described for MoS_2. The IX contrast evolution as a function of demodulation order highlights the sensitivity of s-SNOM to local dielectric variations.

RAMAN SPECTRA

This section shows the Raman spectra of the sample sites, showing that the MoS_2 is indeed four layers and that the heterostructure, Fig. S6(a), shows both A_{1g} modes of each respective monolayer that comprises it. The separation of $25\,\mathrm{cm}^{-1}$ between the modes in MoS_2, Fig. S6(a), indicates that the slab is indeed four layers [7].

In the heterostructure, you can see the out-of-plane A_{1g} modes for both $MoSe_2$ at

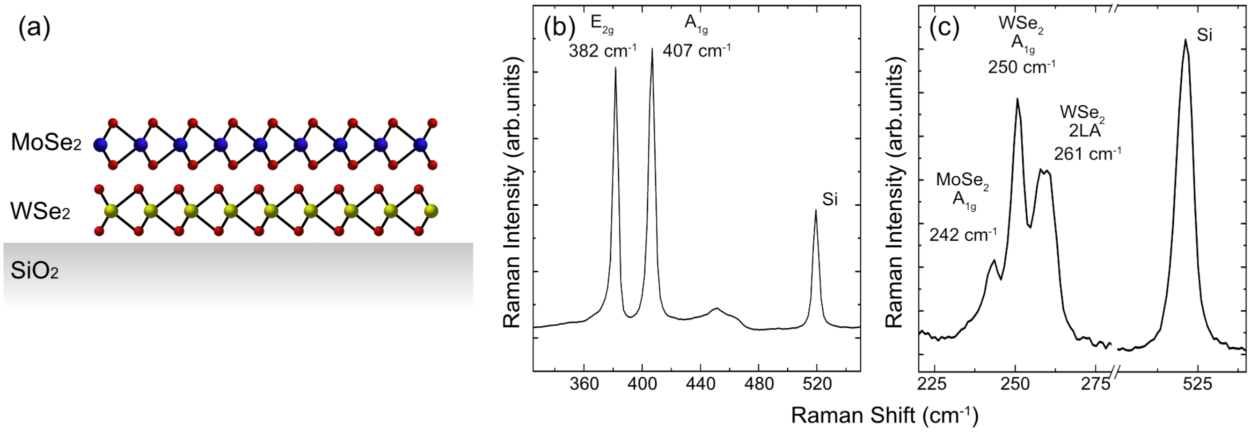

FIG. S6. Sample configuration and Raman spectra confirming the composition of the investigated samples. (a) $MoSe_2/WSe_2$ HT sample illustration (b) The spectrum of four-layer MoS_2 (4L-MoS_2) shows the characteristic E_{2g} (382 cm^{-1}) and A_{1g} (407 cm^{-1}) modes, with a separation of 25 cm^{-1}, consistent with a four-layer thickness. (c) The spectrum of the $MoSe_2/WSe_2$ heterostructure (HT) features the $MoSe_2$ A_{1g} mode (242 cm^{-1}) and the characteristic WSe_2 A_{1g} (250 cm^{-1}) and 2LA (261 cm^{-1}) modes, confirming the presence of both materials. The Si peak ($\sim$520 cm^{-1}) serves as a reference.

242 cm^{-1}, and WSe_2 at 250 cm^{-1}, Fig. S6(b). An additional 2LA mode from WSe_2 is also present at 261 cm^{-1} along with the Silicon peak coming from the substrate. These Raman spectra confirm that the MoS_2 sample is a four-layer slab, as assumed in the manuscript. The $MoSe_2/WSe_2$ heterostructure exhibits well-defined monolayer A_{1g} modes, indicating that the layers are sufficiently coupled for their Raman intensity to be comparable to that of the silicon substrate.

REFERENCES

[1] A. Cvitkovic, N. Ocelic, and R. Hillenbrand. Analytical model for quantitative prediction of material contrasts in scattering-type near-field optical microscopy. *Optics Express*, **15**, 8550 (2007).

[2] B. Hauer, A. P. Engelhardt, and T. Taubner. Quasi-analytical model for scattering infrared

near-field microscopy on layered systems. *Optics Express*, **20**, 13173 (2012).

[3] L. Mester. Substrate-enhanced and subsurface infrared near-field spectroscopy of organic layers. Ph.D. thesis, Universidad del País Vasco/Euskal Herriko Unibertsitatea (2021).

[4] Y. Jiang, S. Chen, W. Zheng, B. Zheng, and A. Pan. Interlayer exciton formation, relaxation, and transport in TMD van der Waals heterostructures (2021).

[5] A. Splendiani, L. Sun, Y. Zhang, T. Li, J. Kim, C.-Y. Chim, G. Galli, and F. Wang. Emerging photoluminescence in monolayer mos_2. *Nano Letters*, **10**, 1271–1275 (2010).

[6] N. Ocelic, A. Huber, and R. Hillenbrand. Pseudoheterodyne detection for background-free near-field spectroscopy. *Applied Physics Letters*, **89**, 101124 (2006).

[7] C. Lee, H. Yan, L. E. Brus, T. F. Heinz, J. Hone, and S. Ryu. Anomalous Lattice Vibrations of Single- and Few-Layer MoS 2. *ACS Nano*, **4**, 2695–2700 (2010).
