# Supplementary material for: Characterizing Interlayer Excitons by Spectral Signature in Scattering Visible Near-Field Microscopy
Source: J Phys Chem Lett. 2025 Jun 30;16(27):6960–7. doi: 10.1021/acs.jpclett.5c01052 (PMC12257585; doi:10.1021/acs.jpclett.5c01052)
Supplement: Supplementary file 2 [file jz5c01052_si_002.pdf]

jz-2025-01052b.R1

Name: Peer Review Information for "Characterising Interlayer Excitons by Spectral Signature in Scattering Visible Near-Field Microscopy"

First Round of Reviewer Comments

Reviewer: 1

Comments to the Author

Garrity and co-authors report on s-SNOM investigations in van der Waals heterostructures. In this study the optical response was determined from spatial averaging amplitude and phase images for varying laser energies and compared to model calculations. The authors first tested their approach on a defective 4-layer MoS_2 tuning the laser energy across the B exciton resonance. They then investigated a $MoSe_2/WSe_2$ heterostructure sample for which they first identified the formation of interlayer excitons from PL spectra. The obtained spectrally resolved S-SNOM data revealed the interlayer exciton resonance demonstrating the sensitivity of s-SNOM detection enabling the observation of the weak response associated with interlayer excitons.

In conclusion, the present manuscript reports on important achievements that warrant publication in this journal. Prior to publication, several points need to be addressed and clarified, however.

1. The authors highlight that the introduction of disorder using ion-irradiation allows them to study how inhomogeneous broadening affects exciton behavior. However, no data is shown for pristine samples for comparison that would be needed to show such an influence. Moreover, the deduced linewidth of the disordered 4L-MoS_2 sample appears to be on the lower side of those reported in literature. The statement on page 6 that defect-assisted recombination was a dominant broadening mechanism is not supported well by the shown data given a total non-radiative linewidth of 10 meV, which is half the radiative width. To substantiate the discussion on the influence of disorder I recommend including the corresponding s-SNOM data of a pristine sample in the supplementary material.

2. Color scales need to be shown in Fig. 2 (a), (b) and (d), (e) to help the reader in assessing the observed spatial and spectral contrast. The largest contrast is observed in (e) at 2.14 eV but there is only a weak signal seen in (f) at this energy. Could (d) and (e) have been exchanged? This would also be consistent with the images shown in supplementary Figure 2(b) and (e) recorded at 2 eV.

3. Color scales are also needed for Figure 4 together with an illustration of the lateral sample composition. Here, the SiO_2 substrate appears to be seen on the left side. If the color scale is the same as the one included in the SI, the phase image seems to show ~0.1 for the left side (SiO_2?) whereas the heterostructure area appears to show more red points on average indicating values below 0? Is this consistent with the discussion? To clarify this, the authors need to mark the area from which the spatially averaged amplitudes and phases shown in Fig. 4 (c) and (f) have been obtained.

4. On page 2, the physical unit of the He^+ ion beam diameter is missing.

Reviewer: 2

Comments to the Author

Summary:

This work presents the use of s-SNOM to resolve intra- and interlayer excitons at the nanoscale, enabling access to their spectral features and transition energies. The authors demonstrate that s-SNOM can directly probe the near-field optical response of interlayer excitons by extracting the complex dielectric function, offering a promising route for investigating the optical signatures and excitonic dynamics in transition metal dichalcogenide (TMD) heterostructures. While the study introduces a novel and scientifically valuable approach, the manuscript currently suffers from several methodological, interpretational, and presentation issues. Substantial revision is necessary before the work can be considered for publication in The Journal of Physical Chemistry Letters.

Reviewer Comments:

1. Sample Fabrication

The manuscript mentions the use of an oil bath to remove and "iron out" bubbles in the heterostructure; however, the references cited (12, 18, 19) do not support or discuss this specific method. Furthermore, Figure 3a still shows a visibly uneven surface, which raises questions about the effectiveness of this process. To support the methodology, the authors should consider including before-and-after optical or AFM images to demonstrate the impact of the oil bath treatment. Additionally, were any quantitative surface roughness measurements (e.g., AFM) performed to assess and support the claim of surface uniformity? This is critical, as surface topology could influence the optical measurements in s-SNOM.

2. s-SNOM Spatial Resolution & Tip Specification

The authors claim a spatial resolution of approximately 25 nm in their s-SNOM measurements. This claim should be supported by experimental validation. For example, did the authors map across a known sharp interface (e.g., the heterostructure boundary) to confirm this spatial resolution? Furthermore, what is the specification of the s-SNOM tip used (e.g., brand, tip radius)? Considering that tips may degrade over time, additional details are necessary to support this resolution claim.

3. Fitting Parameters & Ambiguities

The conclusion acknowledges that the fitting parameters—such as oscillator strength and linewidths—are not uniquely determined. While this is reasonable due to model degeneracy, the authors should implement or suggest possible cross-validation strategies to reduce ambiguity. For instance, fitting the same model across different spatial locations, comparing with independent PL or absorption data, or fixing some parameters based on literature values could enhance robustness and reliability.

4. Fit Fitting Accuracy in Figure 2

In Figure 2, the black theoretical fit line slightly deviates from the data points, especially in panel f near ~2.05–2.06 eV. Without prior knowledge of the theory curve, one might infer a local dip at that point. The authors are encouraged to include more data points in that energy region, if possible, to better support the claimed fit and rule out alternative interpretations due to sparse sampling.

5. 1.35 eV Peak Designation in s-SNOM Data

In Figure 3b (PL) and Figure 3d (absorption), there is a noticeable Stokes shift, as shown in the PL peak appearing at a slightly lower energy due to relaxation processes. However, in Figure 4f (phase signal from s-SNOM), the resonance peak appears exactly at the PL peak

energy of 1.35 eV. This raises the question of whether the assignment of the peak energy in the s-SNOM data has been arbitrarily aligned with the PL result. The authors should clarify this point and explain whether the observed alignment is coincidental, expected, or enforced during fitting.

6. Direct vs. Indirect Interlayer Exciton Transition

There appears to be a contradiction between the interpretation of the IX transition in different parts of the manuscript. In Figure 3d, the authors state that no absorption from the IX is seen due to its indirect nature. However, in the description of Figure 4f, they refer to the IX transition as an "almost momentum-direct K–K transition". The manuscript lacks a clear and consistent explanation of whether the interlayer exciton observed is fundamentally a direct or indirect transition. The authors should explicitly discuss the nature of the transition (momentum direct vs. indirect) and how it influences their interpretation of the s-SNOM measurements.

Author's Response to Peer Review Comments:

Freie Universität Berlin
FB Physik

Oisín Garrity
Arnimallee 14
14195 Berlin

Telefon +49 30 838-54294
E-Mail o.garrity@fu-berlin.de
Internet www.fu-berlin.de

08.05.2025

Revision of manuscript jz-2025-01052b

Dear Editor,

Thank you for sending us the positive email on jz-2025-01052b concerning our manuscript "Characterising Interlayer Excitons by Spectral Signature in Scattering Visible Near-Field Microscopy." We thank the reviewers for their work and for helping us to improve the manuscript.

We carefully revised our paper and considered the points raised by the reviewers. We hope that our improvements are satisfactory, and our manuscript can be published in JPCL.

Sincerely,

Oisín Garrity

REVIEWER #1

We thank the reviewer very much for the very good valuation and for highlighting the importance of our results. The comments are helpful to improve our work. In detail we answer to the questions as follows.

The authors highlight that the introduction of disorder using ion-irradiation allows them to study how inhomogeneous broadening affects exciton behavior. However, no data is shown for pristine samples for comparison that would be needed to show such an influence. Moreover, the deduced linewidth of the disordered 4L-MoS2 sample appears to be on the lower side of those reported in literature. The statement on page 6 that defect-assisted recombination was a dominant broadening mechanism is not supported well by the shown data given a total nonradiative linewidth of 10 meV, which is half the radiative width. To substantiate the discussion on the influence of disorder I recommend including the corresponding s-SNOM data of a pristine sample in the supplementary material.

ANSWER:

We thank the reviewer for this insightful comment and agree that the manuscript lacked a direct comparison to pristine material. To address this, we have added two new sections to the Supporting Information:

"Validating the Multilayer FDM on Pristine 1L-MoS_2/hBN" and "4L-MoS_2 Irradiation."

In the first, we present near-field data from a pristine (non-irradiated) monolayer MoS_2 flake on a 10nm hBN substrate. This includes the AFM topography, amplitude images at multiple excitation energies, and the extracted spectral response, which is modelled using the multilayer finite dipole method (FDM). The fit reveals a sharp B-exciton resonance at 1.98eV with a linewidth of 30meV, significantly narrower than the 50meV used for the irradiated 4L-MoS_2 sample in the main text. This comparison confirms that the broader resonance observed in the manuscript arises from disorder-induced damping.

In the second new section, we include photoluminescence (PL) spectra from both pristine and irradiated 4L-MoS_2 regions, along with a PL intensity map highlighting the irradiated area. Voigt-profile fits (not shown) indicate substantial post-irradiation broadening (A-exciton: 293meV; B-exciton: 132meV). Notably, the B-exciton remains detectable as a weak shoulder, and its peak

energy (~2.02eV) closely matches the resonance energy extracted from our nearfield model. Together, these data substantiate the discussion of disorder-induced broadening and support the model parameters used in the manuscript.

We also address the previously reported linewidth at 1200 meV was too small. This prompted us to re-evaluate the fit of the B-exciton in 4L-MoS2. We increased the fitted total linewidth from 30 meV to 50 meV, in closer agreement with the PL spectrum of the irradiated region, which exhibits a FWHM of 132 meV. The amplitude parameter A_B was also adjusted accordingly to maintain a good match to the measured near-field contrast. These updates are reflected in the revised manuscript and Supporting Information.

Pg. 3: To validate our methodology, we first apply it to a pristine monolayer of MoS$_2$ on hBN, where the extracted exciton parameters from s-SNOM closely match those from PL and literature values (see Supporting Information - Fig. S2).

Pg. 3: This procedure successfully retrieves the known optical response of XB^M in MoS2 and establishes a robust basis for extracting dielectric functions from near-field data, while allowing us to explore the impact of disorder and broadening in the multilayer case.

Pg. 6: Photoluminescence (PL) spectroscopy of the same flake shows a clear broadening of the B exciton peak from 30 meV in the pristine region to 132 meV post-irradiation, corroborating the disorder-induced damping inferred from the near-field response.

Pg. 6: We increased the fitted total linewidth from 30 meV to 50 meV, in closer agreement with the PL spectrum of the irradiated region…

Pg. 6: We increased the fitted total linewidth from 30 meV to 50 meV, in closer agreement with the PL spectrum of the irradiated region, which exhibits a FWHM of 132 meV. The amplitude parameter A_B was also adjusted accordingly to maintain a good match to the measured near-field contrast.

Pg. 7: While fitting a Lorentzian model to near-field data can exhibit parameter degeneracy, where multiple combinations of oscillator strength and linewidth yield comparable results, this ambiguity is significantly mitigated by cross-validation with PL spectroscopy. The linewidths and peak energies extracted from the near-field model are consistent with the PL spectra, and the large amplitude parameters required for matching near-field contrast are supported by prior work on resonant nanophotonics in 2D materials.

Pg. 7: ...the large amplitude parameters required for matching near-field contrast are supported by prior work on resonant nanophotonics in 2D materials

Color scales need to be shown in Fig. 2 (a), (b) and (d), (e) to help the reader in assessing the observed spatial and spectral contrast. The largest contrast is observed in (e) at 2.14 eV but there is only a weak signal seen in (f) at this energy. Could (d) and (e) have been exchanged? This would also be consistent with the images shown in supplementary Figure 2(b) and (e) recorded at 2 eV.

ANSWER:

We thank the reviewer for catching this. We have added color scales to Figures 2 and 4 as requested. Upon reviewing the panel assignments, we also found that the order of panels (d) and (e) in Figure

2 was indeed switched in the original version. This has now been corrected so that the panels match their intended excitation energies and the discussion in the text.

Color scales are also needed for Figure 4 together with an illustration of the lateral sample composition. Here, the SiO2 substrate appears to be seen on the left side. If the color scale is the same as the one included in the SI, the phase image seems to show ~0.1 for the left side (SiO2?) whereas the heterostructure area appears to show more red points on average indicating values below 0? Is this consistent with the discussion? To clarify this, the authors need to mark the area from which the spatially averaged amplitudes and phases shown in Fig. 4 (c) and (f) have been obtained.

ANSWER:

We appreciate the reviewer's detailed reading. As requested, we have added color scales to all panels in Figure 4. The lateral structure of the sample, including the position of the heterostructure and SiO_2 substrate, is already described in the manuscript and visible in the AFM image in Fig. 3b. However, for clarity, we now also include an annotated version of the sample layout in the Supporting Information (Fig. S6(a)), along with a brief explanation of how the averaging regions were selected in the same section.

Regarding the apparent negative phase values: the contrast arises from the arbitrary scaling of the pseudo-heterodyne demodulated phase signal, which is normalized such that the reference substrate (SiO_2) defines the zero level. This is a common convention in s-SNOM literature and does not reflect an absolute phase shift. We have added a note to the caption of Fig. 4 to clarify this point.

Fig. 4: (caption): Phase is referenced to SiO_2 using the standard pseudo-heterodyne convention, where only relative phase contrast is accessible.

SI Fig. S6 caption and text: ...we now also include an annotated version of the sample layout in the Supporting Information (Fig. S6(a)), along with a brief explanation of how the averaging regions were selected...

On page 2, the physical unit of the He^+ ion beam diameter is missing.

ANSWER:

We thank the reviewer for catching this oversight. The physical unit has now been added to the description of the He^+ ion beam diameter in the manuscript.

Pg. 2: The flake was irradiated with a He^+ ion beam (diameter 0.5 nm, energy 7.5 keV)...

REVIEWER #2

We thank the reviewer for the positive evaluation of our manuscript. We revised the manuscript and address the comments accordingly after considering the suggestions of the reviewer.

The manuscript mentions the use of an oil bath to remove and "iron out" bubbles in the heterostructure; however, the references cited (12, 18, 19) do not support or discuss this specific method. Furthermore, Figure 3a still shows a visibly uneven surface, which raises questions about the effectiveness of this process. To support the methodology, the authors should consider including before-and-after optical or AFM images to demonstrate the impact of the oil bath treatment. Additionally, were any quantitative surface roughness measurements (e.g., AFM) performed to assess and support the claim of surface uniformity?

ANSWER:

We thank the reviewer for this helpful suggestion. We agree that the description of the annealing procedure and its results required clarification. We have removed the previously cited references and now cite Jain *et al.* (2018), *"Minimizing residues and strain in 2D materials transferred from PDMS"* (2D Mater. **5**, 045019), where a similar vacuum annealing approach is used to reduce bubbles, wrinkles, and surface contaminants.

Regarding the surface morphology shown in Fig. 3a, we now include quantitative roughness data extracted from the active interlayer region. The RMS roughness in this area is 973.7 pm, with an average surface slope of 26.2×10^{-3}. Compared to other reports (e.g., 2–4 nm in Ghiami et al., ACS Appl. Electron. Mater. 2025) this is a comparable RMS. These values indicate a locally flat interface suitable for near-field optical measurements. Although we do not have a "before" AFM scan of the same area (as vacuum annealing was part of standard post-stacking processing), the measured roughness is consistent with successful flattening. These values are now quoted in the Supporting Information. We also include quantitative roughness data extracted from the active interlayer region.

Pg. 4: Following the transfer, the heterostructure was placed in a test tube and which was then submerged in an oil bath at 300 °C for 4 hours at a pressure of 2x107 mbar. This method facilitates the removal of polymer residues and reduces interfacial bubbles through capillary action and thermal relaxation.

Pg. 4: The RMS surface roughness in the active region was measured to be approximately 974pm (surface slope = 26.2×10^{-3}*., Supporting Fig. S5 (b)) [19]*

The authors claim a spatial resolution of approximately 25 nm in their s-SNOM measurements. This claim should be supported by experimental validation. For example, did the authors map across a known sharp interface (e.g., the heterostructure boundary) to confirm this spatial resolution? Furthermore, what is the specification of the s-SNOM tip used (e.g., brand, tip radius)?

ANSWER:

We thank the reviewer for this valuable observation. We acknowledge that the tip specifications were not clearly stated in the manuscript. Two different tip types were used across the datasets:

- For the 4L-MoS$_2$ measurements, commercially available contamination free **nano-FTIR probes** (attocube, nominal tip radius ~50 nm) were used.
- For the 1L-MoS$_2$ and MoSe$_2$/WSe$_2$ heterostructure measurements, **PtIr-coated Arrow-NCPt tips** from NanoWorld were used, with a nominal tip radius of ~20 nm.

These details are now clarified in the manuscript.

We have also added a reference to our prior work using the same s-SNOM system:
Kusch et al., "Double Tips for In-Plane Polarized Near-Field Microscopy and Spectroscopy," Nano Lett. (2020), where a spatial resolution via near-foield moicroscopy, spectroscopy and AFM of below 50 nm was validated by scanning across a sharp interface. This work supports the stated resolution and tip performance in our current study.

Pg. 4: The 4L-MoS2 was samples were analyzed using a dual s-SNOM system [11, 20] (NeaSNOM from attocube systems AG, Germany) integrated with nano-FTIR tips (attocube systems AG) that had an apex radius ≈ 50nm and a resonance frequency in the range 240-380 kHz. For 1L-MoS2 and the MoSe2/WSe2 heterostructure, PtIr-coated tapping-mode tip (NanoWorld Arrow-NCPt, nominal radius ≈ 20 nm) was used. The resolution of these nearfield probes have been revealed in previous work to be 20nm.

<u>*The conclusion acknowledges that the fitting parameters—such as oscillator strength and linewidths—are not uniquely determined. While this is reasonable due to model degeneracy, the authors should implement or suggest possible cross-validation strategies to reduce ambiguity. For instance, fitting the same model across different spatial locations, comparing with independent PL or absorption data, or fixing some parameters based on literature values could enhance robustness and reliability.*</u>

ANSWER:

We thank the reviewer for this helpful suggestion. We agree that cross-validation is important for increasing confidence in the extracted dielectric function parameters. To this end, we have added photoluminescence (PL) data from both pristine and irradiated regions of the 4L-MoS_2 flake, as well as from a pristine 1L-MoS_2 sample, to the Supporting Information.

The PL spectra are compared directly to the near-field model fits presented in the manuscript and SI. Notably, the B-exciton in the irradiated 4L-MoS_2 region exhibits a FWHM of ~130 meV, significantly broader than the 50 meV used in the near-field model. This is consistent with PL being sensitive to additional broadening mechanisms, including phononassisted transitions, inhomogeneous strain, and disorder-related recombination, that are less pronounced in s-SNOM. We also attempted to fit the s-SNOM data using the PL-derived linewidths directly but found that such values overestimated the damping in the near-field response. This highlights the need for technique-specific modelling but supports the overall trend of irradiation-induced broadening.

These comparisons help validate the resonance energies and relative linewidths extracted from our fits. While PL offers valuable insight, we agree that further constraining model parameters with

complementary techniques such as ellipsometry or transport spectroscopy would be beneficial in future studies. Ellipsometry could, in principle, yield values for the exciton energy and total broadening, but typically requires large, uniform sample areas and more complex multilayer modelling. Transport measurements may also access electronic linewidths indirectly via mobility or scattering rates, but are challenging to perform on spatially heterogeneous samples like ours.

Pg. 9: Photoluminescence (PL) spectroscopy of the same flake shows a clear broadening of the B exciton peak from 30meV in the pristine region to 132 meV post-irradiation, corroborating the disorder-induced damping inferred from the near-_eld response (See Supporting information - Fig. S3, Table S2).

Pg. 15: While fitting a Lorentzian model to near-field data can exhibit parameter degeneracy, where multiple combinations of oscillator strength and linewidth yield comparable results, this ambiguity is significantly mitigated by cross-validation with PL spectroscopy. The linewidths and peak energies extracted from the near-field model are consistent with the PL spectra, and the large amplitude parameters required for matching near-field contrast are supported by prior work on resonant nano-photonics in 2D materials.

In Figure 2, the black theoretical fit line slightly deviates from the data points, especially in panel f near ~2.05–2.06 eV. Without prior knowledge of the theory curve, one might infer a local dip at that point. The authors are encouraged to include more data points in that energy region, if possible, to better support the claimed fit and rule out alternative interpretations due to sparse sampling.

ANSWER:

We thank the reviewer for this close observation. We acknowledge that the spectral sampling around 2.05–2.06 eV is relatively sparse, which may give the appearance of a dip in Fig. 2f that is not strongly supported by adjacent data points. However, we emphasize that this feature is not forced into the model. Rather, it arises from the dielectric response calculated via the multilayer finite dipole model, which simultaneously fits both the amplitude and phase contrast over the full spectral range.

Unfortunately, the original sample is no longer available, and the tunable laser used in our experiments does not permit energy steps below ~7 nm, limiting our ability to add finer spectral resolution in this region.

To further support the validity of our approach, we also applied the same model to a pristine monolayer MoS_2 flake on hBN, using parameters extracted from PL and literature. The model reproduced the near-field response in that system with excellent agreement (see Supporting Information, Fig. S2), lending confidence to the modelling framework and its ability to recover real dielectric features.

Pg. 3: To validate our methodology, we first apply it to a pristine monolayer of MoS2 on hBN, where the extracted exciton parameters from s-SNOM closely match those from PL and literature values (see Supporting Information - Fig. S2).

Pg. 6: To establish the reliability of our method, we first apply it to a well-characterized fourlayer MoS2 slab, Fig. 1(a), where the XBM exciton provides a strong and well-defined spectral feature in the visible range. In parallel, we validate the multilayer dielectric model itself using pristine monolayer MoS2 on hBN, where the XBM lineshape is sharp and well-documented (see Supporting Information Fig S2, Table S1). These benchmarks allow us to validate both the optical model and our s-SNOM acquisition and processing strategy, and to explore the inuence of defects on the exciton response, before turning to the more challenging case of IXs in MoSe2/WSe2 heterostructures.

Pg. 9: To validate the accuracy of this approach, we first applied the multilayer FDM model to a pristine 1L-MoS2 flake on 10nm of hBN. The resulting fit reproduced the near-field contrast across the B-exciton resonance with excellent agreement to the PL spectrum and literature values (see Fig. S2 and Table S1 - Supporting Information), establishing a reliable reference point for analysis of more complex systems.

In Figure 3b (PL) and Figure 3d (absorption), there is a noticeable Stokes shift, as shown in the PL peak appearing at a slightly lower energy due to relaxation processes. However, in Figure 4f (phase signal from s-SNOM), the resonance peak appears exactly at the PL peak energy of 1.35 eV. This raises the question of whether the assignment of the peak energy in the s-SNOM data has been arbitrarily aligned with the PL result. The authors should clarify this point and explain whether the observed alignment is coincidental, expected, or enforced during fitting.

ANSWER:

We thank the reviewer for this thoughtful observation. The apparent discrepancy arises because the absorption and s-SNOM measurements were performed on heterostructures supported by different substrates. Specifically, the absorption spectrum shown in Fig. 3d was measured from a sample on a sapphire substrate, while the PL and s-SNOM data in Figures 3b and 4 were acquired from a heterostructure on a SiO_2/Si substrate.

This substrate difference likely contributes to the observed Stokes shift, as the surrounding dielectric environment can influence the exciton binding energy and relaxation dynamics. The alignment between the s-SNOM phase resonance and the PL peak energy in our main sample (on SiO_2) is therefore physically consistent and not artificially enforced in the fitting. We have clarified this point in the caption of Fig. 3 and in the main text.

Pg. 11: We note that the absorption spectrum was measured on a $MoSe_2/WSe_2$ heterostructure on sapphire, whereas the PL was acquired on a similar heterostructure on a SiO2 substrate.

There appears to be a contradiction between the interpretation of the IX transition in different parts of the manuscript. In Figure 3d, the authors state that no absorption from the IX is seen due to its indirect nature. However, in the description of Figure 4f, they refer to the IX transition as an "almost momentum-direct K–K transition". The manuscript lacks a clear and consistent explanation of whether the interlayer exciton observed is fundamentally a direct or indirect

transition. The authors should explicitly discuss the nature of the transition (momentum direct vs. indirect) and how it influences their interpretation of the s-SNOM measurements.

ANSWER:

We thank the reviewer for identifying this contradiction. In the original sentence, we intended to convey that the interlayer exciton (IX) becomes optically active and detectable via PL as the twist angle approaches 0° or 60°, due to improved overlap and reduced momentum mismatch. However, the phrasing mistakenly implied that the IX is momentum-direct (K–K), which is not generally the case for $MoSe_2/WSe_2$ heterostructures.

We have revised the relevant paragraph in the manuscript (associated with Fig. 4f) to avoid this confusion and now explicitly state that the IX is typically momentum-indirect (e.g., K–Q), consistent with its absence in absorption and weak but detectable PL emission. Additionally, we add a citation that highlight that the heterostructures discussed here form a type-2-band alignment, Jiang et al., Light: Science & Applications 10, 72 (2021).

Pg. 14: The transition energy of the interlayer exciton (IX), extracted from the fit, ω_0 = 1.35 meV agrees with the PL peak in Fig. 3(a). Although the heterostructure has a near-zero twist angle, the IX is expected to be momentum-indirect (e.g., K-Q), consistent with its weak optical activity. The extracted non-radiative linewidth, Γ_{nr}= 15 meV, is in line with prior reports for such transitions, while the radiative linewidth, Γ_r= 5 meV, reflects the suppressed oscillator strength typical of spatially indirect interlayer excitons.

jz-2025-01052b.R2

Name: Peer Review Information for "Characterising Interlayer Excitons by Spectral Signature in Scattering Visible Near-Field Microscopy"

Second Round of Reviewer Comments

Reviewer: 1

Comments to the Author

In the revised manuscript and their point-by-point response, the authors have addressed all the previously raised concerns. I recommend accepting the manuscript in its present form.

Author's Response to Peer Review Comments:

Freie Universität Berlin
FB Physik

Oisín Garrity
Arnimallee 14
14195 Berlin

Telefon +49 30 838-54294
E-Mail o.garrity@fu-berlin.de
Internet www.fu-berlin.de

20.06.2025

Revision of manuscript jz-2025-01052b.R1

Dear Editor,

We are delighted to hear your decision regarding the manuscript. We thank the reviewers for their constructive feedback, which significantly improved the work overall. We have now addressed the remaining editorial points outlined in your recent email, including adding the TOC graphic to the end of the manuscript and correcting the Supporting Information description in the main text.

We hope the revised files now meet all editorial requirements and are happy to provide anything further if needed.

Sincerely,

Oisín Garrity
